# Women's Empowerment, WaSH, and Child Feeding: Implications for Childhood Stunting in South Asia

**DOI:** 10.1111/mcn.70223

**Published:** 2026-07-17

**Authors:** Saira Parveen Jolly, Regien Biesma, Robert Lensink, Md. Khayrul Bhuyan, Kaosar Afsana, Jurjen van der Schans

**Affiliations:** ^1^ Department of Health Sciences, Global Health Unit University Medical Center Groningen Groningen the Netherlands; ^2^ BRAC James P Grant School of Public Health BRAC University Dhaka Bangladesh; ^3^ University Medical Center Utrecht, Julius Center Global Health University of Utrecht Utrecht the Netherlands; ^4^ Department of Economics, Econometrics and Finance University of Groningen Groningen the Netherlands

**Keywords:** decision‐making, food, nutrition, South Asia, stunting, WaSH, women's empowerment

## Abstract

Despite socio‐economic progress, prevalence of childhood stunting remains high in South Asia—a phenomenon often referred to as the “Asian Enigma.” This paradox stems from suppressed women's empowerment, poor water, sanitation, and hygiene (WaSH) conditions and inadequate child‐feeding. Evidence shows that women's empowerment improves these underlying factors associated with improved stunting. We examined how domains of women's empowerment relate to the risk of childhood stunting in South Asia directly and indirectly in interaction with household improved WaSH facility and age‐appropriate food consumption. Using recent Demographic and Health Survey data from Bangladesh, India, Nepal, and Pakistan, we analyzed a sample of 37,620 married women, aged 15–49 years, currently cohabiting with husbands and having an under‐five child. We constructed three domains of survey‐based women's empowerment index‐global: social independence, intrinsic agency, and instrumental agency, by confirmatory factor analysis, using generalized structural equation modelling. We developed three different models by using generalized linear mixed‐effects models with robust error variance to estimate the relative risk (RR) of stunting with 95% confidence interval (CI). We found social independence [RR: 0.86, 95% CI: 0.83, 0.90], intrinsic agency [0.98 (0.97, 0.99)] and instrumental agency [0.99 (0.98, 0.99)] were associated with reduced risk of stunting in South Asia. Though interaction between social independence and WaSH was associated with reduced risk [0.92 (0.86, 0.99)], interaction between social independence [1.12 (1.07, 1.17)] and instrumental agency [1.03 (1.01, 1.05)] and food consumption increased risk of stunting in South Asia. Interactions between women's empowerment and WaSH and food consumption reveal complex effects including both synergy and trade‐offs, highlighting the need for integrated strategies combining women's empowerment, WaSH improvements, and targeted nutrition specific interventions in South Asia.

## Summary

- Women's empowerment is associated with a lower risk of childhood stunting, independent of other factors.
- The combination of women's empowerment and improved household WaSH conditions is associated with a lower risk of stunting.
- However, when women's empowerment is considered together with age‐appropriate food intake, it appears to be associated with a higher risk of stunting, highlighting critical gaps in child nutrition.

## 1 Introduction

1.1

Stunting remains a critical global health challenge, affecting an estimated 148 million children in 2023 (UNICEF/WHO/World Bank Group 2023). South Asia bears the highest regional prevalence (53.7%), exceeding other parts of Asia and ranking second globally. The annual reduction rate (2.8%) in this region is insufficient to meet the Sustainable Development Goal (SDG) 2.2 target of ending all forms of malnutrition by 2030 (UNICEF/WHO/World Bank Group 2023). Stunted children, whose height‐for‐age Z (HAZ) score reflects cumulative linear growth retardation, are at an increased risk of impaired cognitive development, lower educational attainment and economic productivity, and chronic disease and mortality (Leroy and Frongillo 2019).

Evidence indicates that the high prevalence of stunting in South Asia is closely linked to inadequate nutrition for women and children during the first 1000 days, from conception to a child's second birthday. During pregnancy, insufficient micronutrient intake increases the risk of maternal anemia, preterm birth, low birthweight, and poor pregnancy and delivery outcomes (Hofmeyr et al. 2018; Makrides and Crowther 2001; Martinat et al. 2021; Zimmermann 2012; Ota et al. 2015; Holick 2019). After birth, suboptimal breastfeeding, inappropriate complementary feeding, low dietary diversity, and inadequate meal frequency increase the risk of growth retardation (Abdulla et al. 2023; Kim et al. 2017; Leroy et al. 2014; Victora et al. 2010). In addition, limited access to improved water, sanitation, and hygiene (WaSH) contributes to recurrent infections and adversely affects healthy growth trajectories (Girma et al. 2024; Wolf et al. 2022).

While South Asia has seen notable improvements in many social indicators, progress in reducing child malnutrition remains slow. This paradox is often referred to as the “Asian Enigma” (Carlson et al. 2015; Ramalingaswami et al. 1996; Rao 2020). Beyond immediate determinants of stunting, entrenched patriarchal norms in South Asia perpetuate undernutrition. These norms limit women's agency through structural barriers such as son preference, early marriage, labor market exclusion, and gender‐based violence (Rao 2020). Across the South Asian region, these constraints are reinforced by sociocultural contexts. For example, purdah in Bangladesh, caste‐ and region‐based inequalities in India, and limited autonomy in rural Pakistan shape women's opportunities and choices (Bushra and Wajiha 2015; Parveen 2007; Rahman 2020). In Nepal, extended family systems are linked to the disempowerment of daughters‐in‐law, while male migration introduces complex dynamics for women's autonomy. Caste and ethnicity further influence empowerment, as norms around mobility, work, and marriage play a crucial role (Doss et al. 2022). These persistent forms of gender inequality slow progress not only toward SDG 2 (Zero Hunger), but also toward several interconnected development goals (Rao 2020). Achieving gender equality and empowering women (SDG 5) is both a marker of social progress and a necessary driver for wider development outcomes. Empowered women are pivotal for reaching targets in zero hunger, gender equality, and clean water (Wei et al. 2021; Quisumbing et al. 2021; Cunningham et al. 2019).

Empowerment, as conceptualized by Kabeer (1999) involves access to resources, agency, and achievements while Sen defines resources and agency together as “capability,” the ability to achieve valued functioning (Sen 1980). Agency encompasses both personal and relational dimensions. Personal agency, often termed intrinsic agency or “power within,” reflects the meaning, motivation, and internal capacities individuals bring to their actions, including self‐efficacy and self‐esteem (Mosedale 2005; Miedema et al. 2018; Huis et al. 2020). When intrinsic agency translates into decision‐making authority (power to'), it is conceptualized as instrumental agency, commonly measured through women's participation in household (HH) decision‐making (Kabeer 1999; Salinger et al. 2024). In contrast, relational agency (power over') refers to the ability to influence the behavior of others, such as partners or family members (Navarro‐Mantas et al. 2022).

Despite growing evidence linking women's empowerment with childhood stunting in South Asia, a critical gap remains in understanding how distinct domains, such as social independence, intrinsic agency, and instrumental agency interact with stunting across multiple countries (Shroff et al. 2009; Paul and Saha 2022; Lakhdir et al. 2024). The instrumental agency has been consistently associated with improved child nutrition; the contributions of social independence and intrinsic agency to reducing stunting remain underexplored and may offer further insights into the persistence of the “Asian Enigma” (Cunningham et al. 2015).

Therefore, this study aims to address this gap by examining how different dimensions of women's empowerment, both directly and indirectly via age‐appropriate food intake and access to improved WaSH facility, influence childhood stunting. We hypothesized that women's empowerment not only has a direct association with lower risk of stunting but also exerts indirect associations through interacting with HH improved WaSH facility and adequate food consumption in the under‐five children.

## 2 Methods

### 2.1 Data Source

We pooled and analyzed the most recent Demographic and Health Survey (DHS) data from Bangladesh (2017–18), India (2019–21), Nepal (2022), and Pakistan (2017–18). We selected four, out of eight South Asian nations based on the availability of complete data required to measure women's empowerment domains and anthropometric indices for both women and children. Details of the DHS data collection procedures have been published elsewhere (NIPORT 2024; IIPS 2021; MoHP 2023; NIPS 2019). For the current analysis, women were selected according to the following inclusion criteria:

- Women with complete data to construct the survey‐based women empowerment index (SWPER)‐global.
- Women who were married and living with their husbands during the survey.
- Women aged 15–49 years with at least one living under‐five child.

Additionally, we excluded children with anthropometric z‐scores ±six times of the standard deviations (SDs) from the mean as these values were flagged as invalid (Croft et al. 2023).

### 2.2 Primary Outcomes

The primary outcome was stunting of offspring, assessed using the WHO (2006) growth standards. Children with HAZ‐scores below minus two SDs from the WHO reference median were classified as stunted.

### 2.3 Exposures

The primary exposures were the three domains of women's empowerment from the SWPER‐global, HH WaSH facility and age‐appropriate food intake by under‐five children.

### 2.4 SWPER‐Global

The three domains of SWPER‐global are social independence, intrinsic agency, and instrumental agency (Ewerling et al. 2020). Internal validity of this measure was established using principal component analysis to derive domain‐specific factor loadings across 62 low‐ and middle‐income countries, while external validation showed strong correlations with the Gender Development Index and Gender Inequality Index, both reflecting progress towards SDG 5 (Ewerling et al. 2020). Despite contextual differences, South Asia and sub‐Saharan Africa exhibit similarly low scores across all three domains (Ewerling et al. 2020). Social independence includes education, access to information, age at first childbirth and cohabitation, and spousal age and education differences. Intrinsic agency captures women's attitudes towards wife‐beating across five scenarios. Instrumental agencies reflect decision‐making autonomy in healthcare, major HH purchases, and visits to family or relatives.

### 2.5 Composite Variables

Two composite variables were constructed: (1) HH WaSH facility, combining improved water sources and sanitary latrines; and (2) age‐appropriate food consumption, including exclusive breastfeeding, meal frequency, and dietary diversity across age groups (Appendix 3; NIPORT 2024; WHO and UNICEF 2021). To include all children in a single variable, two age‐specific definitions of dietary intake were applied (Appendix 3). Infant and young child feeding indicators for under‐two children were based on UNICEF recommendations (UNICEF 2021), while dietary diversity for children aged 24–59 months followed DHS definitions (NIPORT 2024).

### 2.6 Covariates

This study incorporates covariates that establish risk factors for offspring stunting, such as the employment situation of women, sex of the children, place of residence, maternal height, and weight‐for‐age Z (WAZ) and weight‐for‐height Z (WHZ) scores of the children.

### 2.7 Covariates Selection

Covariates were selected based on both theoretical reasoning and multicollinearity diagnostics. A directed acyclic graph (DAG), developed from existing literature on determinants of childhood stunting in South Asia, guided the identification of a minimal sufficient adjustment set (Appendices 1 and 2). The DAG specified hypothesized relationships between women's empowerment (SWPER‐global domains), mediators (child feeding practices and WaSH), socioeconomic and maternal characteristics, and stunting. Additional bivariate and Poisson regression analyses were conducted using DHS variables to inform covariate selection (Appendices 3–5). Multicollinearity was assessed using the variance inflation factor (VIF), with VIF < 5 indicating no substantial collinearity. All covariates were initially included in the model, however, some were excluded due to collinearity. Maternal height and wealth index showed high collinearity with maternal BMI. After excluding BMI, further collinearity emerged between wealth index and HH WaSH facility, and between age‐appropriate food consumption and child's age. Since the study focused on women's empowerment, WaSH, and child nutrition, wealth index and child's age were excluded, while WaSH and age‐appropriate food consumption were retained. In the Pakistan‐specific model, maternal height exhibited high multicollinearity (VIF > 5) and was excluded. Finally, the forward selection method was applied as a model refinement strategy to retain variables based on statistical significance while improving model parsimony. Furthermore, sensitivity analyses including all covariates were conducted; in this full model, WaSH and age‐appropriate food intake were not significantly associated with stunting, and the association direction differed from our final model (Appendix 6).

### 2.8 Data Analysis

Initially, sampling weights were constructed using the primary sampling unit and strata information from the DHS data. These weights were applied in descriptive analyses and in the generalized linear mixed‐effects models (GLMM). Descriptive analyses were conducted to examine differences in factors associated with the three SWPER global domains across countries and by nutritional status. Categorical variables were reported as frequencies and percentages, whereas continuous skewed data were presented as median (interquartile range, IQR).

Confirmatory factor analysis (CFA) was conducted using generalized structural equation modeling (GSEM) to derive three latent constructions: social independence, intrinsic agency, and instrumental agency. We used GSEM because it integrates elements of CFA, while providing a flexible framework for modeling complex relationships among both latent and observed variables (Muthén 1984). Factor loadings for CFA are given in Appendix 7. Model fit was assessed using the Akaike Information Criterion (AIC) and Bayesian Information Criterion (BIC), which are commonly used to compare competing models and identify the most parsimonious model. Lower AIC and BIC values indicate better relative model fit while accounting for model complexity. Because these indices are not standardized on a 0–1 scale, no universal cut‐off values exist; rather, the model with the lowest AIC and BIC is generally considered preferable (Hooper et al. 2008).

For the primary outcome, a GLMM with robust error variance was applied, incorporating sampling weights. A Poisson distribution with log link was selected to estimate the relative risk (RR) of stunting, as odds ratios from cross‐sectional data may overestimate risk (McNutt 2003). This approach provides more reliable estimates of RR (Zou 2004; Chen et al. 2018). Random effects accounted for variability across the four countries, while fixed effects were used for predictors (Gurka et al. 2012). Model 1 tested hypothesis 1, assessing whether higher levels of women's empowerment across domains were associated with a lower risk of stunting. Model 2 tested hypothesis 2, examining whether the interaction between women's empowerment domains and improved HH WaSH facility reduced the risk of stunting among under‐five children. Model 3 tested hypothesis 3, evaluating the interaction between women's empowerment domains and age‐appropriate food intake by under‐five children. The equations for the three models are provided in Appendix 8. Results were reported as adjusted (adj) RR with 95% confidence interval (CI) and *p*‐values. Model fit for Poisson regression was assessed using Pearson and deviance χ² tests, with non‐significant results (*p* > 0.05) indicating adequate fit. Additionally, AIC and BIC were used to compare competing models. Analyses were performed in Stata version 17.0 (StataCorp, College Station, TX, USA).

### 2.9 Ethics Statement

Ethical clearance of this research was obtained from the Institutional Review Board of the BRAC James P Grant School of Public Health, BRAC University, Bangladesh. Reference: IRB protocol NO: IRB‐11June'23‐021.

## 3 Results

Initially, the pooled dataset included 1,400,186 individuals from four countries. After applying all filtering criteria, the final sample comprised 37,620 individuals: 6371 from Bangladesh, 26,398 from India, 1539 from Nepal, and 3412 from Pakistan. Both pooled and country‐specific data provided sufficient statistical power to estimate the risk of stunting reliably (NIPORT 2024; IIPS 2021; MoHP 2023; NIPS 2019.

### 3.1 Nutritional Status of Offspring

We observed that the prevalence of stunting ranged from 32% to 39% across countries, with Pakistan exhibiting the highest prevalence of stunting. Similarly, the prevalence of underweight ranged from 22% to 29%, and wasting ranged from 7% to 18%, both of which were pronounced in India and the lowest was in Nepal (Appendix 9).

### 3.2 Factors Associated With Three Domains of the SWPER‐Global

We stratified the children by their stunting status and compared the distribution of 14 factors associated with the three domains of the SWPER‐global for South Asia overall and by country. Overall, in South Asia approximately one‐fourth of women with non‐stunted children read newspapers. They had an average of eight years of schooling, experienced their first childbirth at 20 years of age, and began cohabiting at 18 years of age (Table 1). The spousal age difference was four years, with no difference in years of schooling between spouses. Newspaper readership of women was highest in India (29% vs.21%) and lowest in Bangladesh (12.2% vs. 6.3%), showing similar patterns across countries between mothers of stunted and non‐stunted children. Women with non‐stunted children had higher years of schooling—nine years in India and Nepal, compared to seven years in Bangladesh and five years in Pakistan. Age at first childbirth and cohabitation was highest in India and lowest in Bangladesh. Spousal age gap was greatest in Bangladesh, though educational differences were similar across the region (Table 1).

**Table 1:** Comparison of factors associated with social independence according to stunting status among under‐five children in South Asia and the four study countries (weighted).

Factors	Pooled		Bangladesh		India		Nepal		Pakistan	
	*N* = 37620		*N* = 6271		*N* = 26398		N = 1539		N = 3412	
	HAZ		HAZ		HAZ		HAZ		HAZ	
	≥−2.00 SD	<−2.00 SD	≥−2.00 SD	<−2.00 SD	≥−2.00 SD	<−2.00 SD	≥−2.00 SD	<−2.00 SD	≥−2.00 SD	<−2.00 SD
	***n*** = **24412**	***n*** = **13205**	***n*** = **4245**	***n*** = **2010**	***n*** = **16928**	***n*** = **9365**	***n*** = **1105**	***n*** = **434**	***n*** = **2060**	***n*** = **1339**
Social independence										
Reading newspapers, %(n)	25.28 (6172)	18.07 (2386)	12.23 (519)	6.32 (127)	29.67 (5022)	21.89 (2050)	19.28 (213)	13.82 (60)	19.23 (396)	10.40 (139)
Highest yrs of schooling, median (IQR)	8 (7)	7 (10)	7 (4)	5 (4)	9 (7)	8 (10)	9 (6)	5 (9)	5 (10)	0 (0, 5)
Women age at 1st birth, in yrs, median (IQR)	20 (15)	20 (4)	18 (4)	18 (3)	21 (4)	20 (4)	20 (4)	19 (4)	21 (5)	20 (18, 23)
Women age at 1st cohabitation, in yrs, median (IQR)	18 (5)	18 (4)	16 (4)	16 (4)	19 (4)	18 (4)	18 (4)	17 (3)	19 (5)	18 (16, 20)
Age difference between husband and wife, in yrs, median (IQR)	−4 (−5)	−4 (−5)	−7 (−6)	−7 (−6)	−4 (−3)	−4 (−4)	−4 (−5)	−3 (−5)	−4 (−6)	−4 (−6)
Years of schooling difference between husband and wife, in yrs, median (IQR)	0 (−5)	0 (−5)	0 (−5)	1 (−4)	0 (7)	0 (4)	0 (4)	−1 (5)	0 (−5)	0 (−5, 0)

Abbreviations: HAZ, height‐for‐age Z score; IQR, interquartile range; Yrs, years.

**Figure 1:**
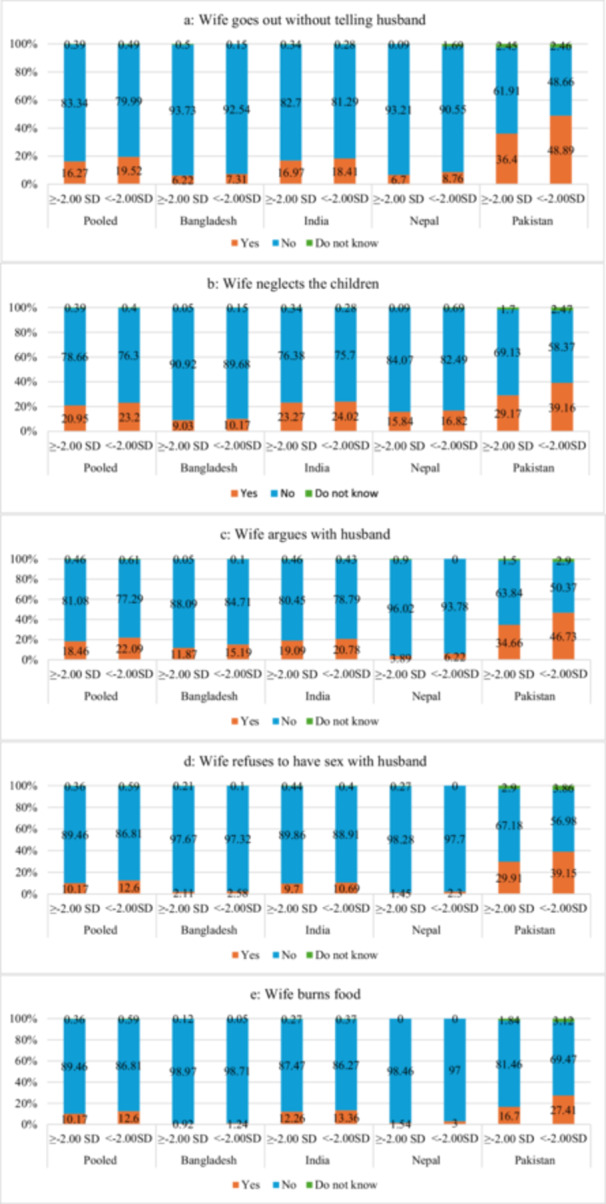
Comparison of factors associated with intrinsic agency according to stunting status among under‐five children in South Asia and the four study countries (weighted).

**Figure 2:**
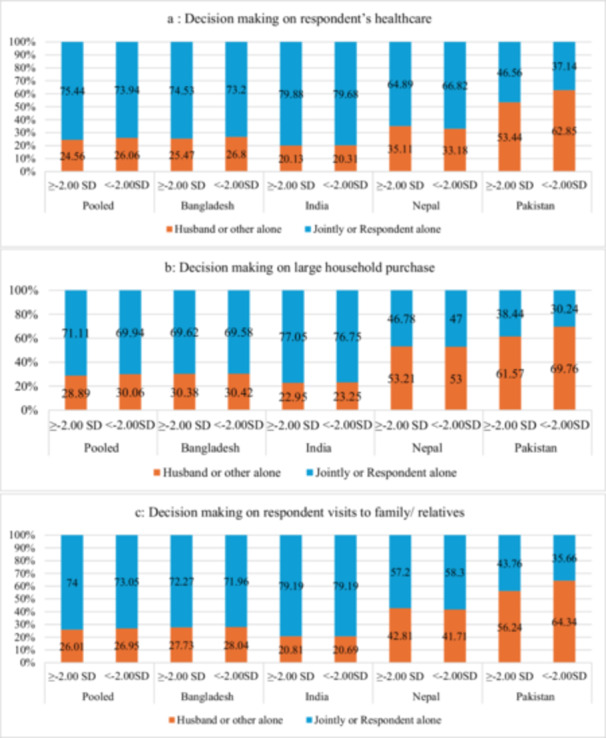
Comparison of factors associated with instrumental agency according to stunting status among under‐five children in South Asia and the four study countries (weighted).

Women with non‐stunted children were less likely to justify wife‐beating. Higher proportions in Bangladesh and Nepal rejected it, while acceptance was most common in Pakistan. Fewer than 10% of women had individual decision‐making autonomy, with higher autonomy in Nepal. Joint decision‐making was common in Bangladesh, India, and Nepal, whereas in Pakistan decisions were mainly husband or HH‐controlled (Figures 1 and 2).

### 3.3 Association Between Stunting and Women's Empowerment (Model 1)

Women's social independence [adj‐RR 0.86 (95% CI 0.83–0.90)] and instrumental agency [0.99 (0.98–0.99)] were associated with reduced stunting risk in South Asia (Table 2 and Appendix 10). In Bangladesh, both social independence [0.82 (0.75–0.90)] and intrinsic agency [0.98 (0.97–0.99)] were associated with reduced risk of stunting. Social independence also had an association with reduced risk of stunting in India [0.88 (0.85–0.92)] and Pakistan [0.75 (0.68–0.83)]. However, in Nepal, none of the SWPER‐global domains had an association with the risk of stunting of offspring (Table 2 and Appendix 10).

**Table 2:** Association between childhood stunting and women's empowerment domains, WaSH, and age‐appropriate food intake after adjusting for covariates (weighted).

Covariates			Countries							
	Pooled		Bangladesh		India		Nepal		Pakistan	
	adj‐RR	95% CI	adj‐RR	95% CI	adj‐RR	95% CI	adj‐RR	95% CI	adj‐RR	95% CI
Model 1										
Social independence	0.86***	0.83–0.90	0.82***	0.75–0.90	0.88***	0.85–0.92	0.92	0.77–1.10	0.75***	0.68–0.83
Intrinsic agency	0.99*	0.99–1.002	0.98**	0.97–0.99	1.00	0.99–1.002	0.97	0.93–1.01	0.99	0.98–1.01
Instrumental agency	0.991**	0.98–0.993	0.99	0.97–1.01	0.99*	0.97–1.00	0.99	0.96–1.03	0.99	0.96–1.01
Age‐appropriate diet (Yes = 1)	0.89***	0.82–0.96	0.87**	0.78–0.97	0.92**	0.86–0.92	1.03	0.71–1.39	0.65***	0.50–0.84
WaSH practice (Yes = 1)	0.90***	0.87–0.93	0.92**	0.86–0.99	0.91***	10.87–0.94	0.72***	0.60–0.87	0.81***	0.73–0.90
Model 2										
Social independence	0.95*	0.90–1.006	0.95	0.84–1.08	0.89***	0.84–0.96	1.16	0.88–1.53	0.92	0.79–1.07
Intrinsic agency	0.98***	0.97–0.99	0.98*	0.96–1.002	0.99	0.98–1.003	0.98	0.91–1.05	1.009	0.78–1.02
Instrumental agency	0.99	0.97–1.01	0.99	0.96–1.02	1.004**	0.98–1.02	1.01	0.93–1.08	1.02	0.97–1.02
WaSH facility (Yes = 1)	0.89***	0.83–0.92	0.83***	0.74–0.92	0.91***	0.88–0.95	0.67***	0.55–0.82	0.71***	0.61–0.83
Social Independence* WaSH	0.92***	0.86–0.99	0.76***	0.64–0.91	0.97*	0.90–1.06	0.72*	0.51–1.02	0.75***	0.61–0.92
Intrinsic agency* WaSH	1.007	0.99–1.01	0.99	0.96–1.02	1.01*	0.99–1.02	0.99	0.91–1.07	0.98	0.95–1.01
Instrumental agency* WaSH	0.98*	0.99–1.03	0.99	0.96–1.03	0.97*	0.95–1.002	0.97	0.94–1.06	0.96	0.90–1.02
Age‐appropriate diet (Yes = 1)	0.87***	0.83–0.92	0.87**	0.78–0.97	0.92**	0.86–0.99	1.002	0.74–1.34	0.66***	0.51–0.85
Model 3										
Social independence	0.85***	0.82–0.89	0.80***	0.73–0.89	0.87***	0.83–0.91	0.89	0.73–1.08	0.74***	0.67–0.83
Intrinsic agency	0.99**	0.99–1.00	0.98	0.93–1.00	1.00	0.99–1.007	0.96	0.93–1.007	1.001	0.98–1.01
Instrumental agency	0.98**	0.98–0.99	0.99	0.97–1.01	0.98**	0.97–0.99	0.99	0.96–1.03	0.99	0.96–1.02
WaSH facility (Yes = 1)	0.90***	0.87–0.93	0.92**	0.86–0.99	0.91***	0.87–0.94	0.72***	0.59–0.87	0.81***	0.73–0.90
Age‐appropriate diet (Yes = 1)	0.89***	0.84–0.95	0.94	0.81–1.08	0.91**	0.85–0.98	0.95	0.68–1.33	0.60***	0.44–0.83
Social Independence* Age‐appropriate diet	1.12**	1.07–1.17	1.16	0.89–1.50	1.13*	0.99–1.30	1.25	0.82–1.90	1.21	0.81–1.82
Intrinsic agency* Age‐appropriate diet	0.98	0.97–1.00	0.99	0.94–1.03	0.99	0.97–1.01	1.08	0.93–1.24	0.92**	0.86–0.99
Instrumental agency* Age‐appropriate diet	1.03**	1.01–1.05	1.02	0.96–1.09	1.03	0.99–1.07	0.96	0.81–1.14	1.04	0.92–1.19

* *p* < 0.1

** *p* < 0.05

*** *p* < 0.01. adjusted relative risk; RR < 1 positive association; RR = 1 no association; RR > 1 negative association; CI ‐ Confidence Interval.

Model 1: HAZ = Social independence + Intrinsic agency + Instrumental agency + Food intake + WaSH + Other covariates.

Pooled: Chi‐square 9807; Prob > chi2 0.000; AIC 45436; BIC 45546; VIF 1.46.

Bangladesh: Chi‐square 2378; Prob > chi2 0.000; AIC 7230; BIC 7318; VIF 1.35.

India: Chi‐square 6585; Prob > chi2 0.000; AIC 32596; BIC 32702; VIF 1.44.

Nepal: Chi‐square 513; Prob > chi2 0.000; AIC 1610; BIC 1679; VIF 1.60.

Pakistan: Bangladesh: Chi‐square 827; Prob > chi2 0.000; AIC 3908; BIC 3967; VIF 1.10.

Model 2: HAZ = social independence + intrinsic agency + instrumental agency + food intake + WaSH + social independence * WaSH + intrinsic Agency * WaSH + Instrumental Agency * WaSH + other covariates.

Pooled: Chi‐square 9889; Prob > chi2 0.000; AIC 45434; BIC 45570; VIF 1.56.

Bangladesh: Chi‐square 2381; Prob > chi2 0.000; AIC 7232; BIC 7339; VIF 1.76. India: Chi‐square 6658; Prob > chi2 0.000; AIC 32597; BIC 32728; VIF 1.54.

Nepal: Chi‐square 519; Prob > chi2 0.000; AIC 1613; BIC 1699; VIF 1.61. Pakistan: Bangladesh: Chi‐square 850; Prob > chi2 0.000; AIC 3905; BIC 3983; VIF 1.45.

Model 3: HAZ = social independence + intrinsic agency + instrumental agency + food intake + social independence * food intake + intrinsic Agency * food intake + Instrumental Agency * food intake + WaSH + other covariates.

Pooled: Chi‐square 9873; Prob > chi2 0.000; AIC 45431; BIC 45567; VIF 4.37.

Bangladesh: Chi‐square 2383; Prob > chi2 0.000; AIC 7235; BIC 7343; VIF 3.25; India: Chi‐square 6624; Prob > chi2 0.000; AIC 32596; BIC 32727; VIF 4.63.

Nepal: Chi‐square 519; Prob > chi2 0.000; AIC 1615; BIC 1700; VIF 5.21; Pakistan: Bangladesh: Chi‐square 828; Prob > chi2 0.000; AIC 3908; BIC 3967; VIF 5.86.

### 3.4 Association Between Stunting and HH Wash Facility and Age‐Appropriate Food Intake by the Children (Model 1)

HH WaSH facility was associated with a reduced risk of stunting in the pooled sample [0.90 (0.87–0.93)] and across countries, including Bangladesh [0.92 (0.86–0.99)], India [0.91 (0.87–0.94)], Nepal [0.72 (0.62–0.89)], and Pakistan [0.81 (0.73–0.90)] (Table 2). Similarly, age‐appropriate food intake was associated with lower risk of stunting in the pooled sample [0.89 (0.82–0.96)] and in Bangladesh [0.87 (0.78–0.97)], India [0.92 (0.86–0.92)], and Pakistan [0.65 (0.50–0.89)], however, not in Nepal (Table 2 and Appendix 10).

### 3.5 Association Between Stunting and Interaction Between Women's Empowerment and HH WaSH Facility (Model 2)

Interactions between women's empowerment domains and HH WaSH facility was included in Model 2 (Table 2 and Appendix 11). We observed that the interaction between social independence and WaSH was associated with a reduced risk of stunting in the pooled South Asian sample [0.92 (0.86–0.99)], as well as in Bangladesh [0.76 (0.64–0.91)] and Pakistan [0.75 (0.61–0.92)] (Table 2 and Appendix 11).

### 3.6 Association Between Stunting and Interaction Between Women's Empowerment and Age‐Appropriate Food Consumption (Model 3)

The interaction between women's empowerment domains and age‐appropriate food consumption by the children was incorporated into Model 3 (Table 2 and Appendix 12). We observed a negative association between a higher risk of stunting and interaction between food intake and social independence [1.12 (1.07–1.17)] and instrumental agency [1.03 (1.01,1.05)] in the South Asian pooled sample. Notably, instrumental agency also showed an independent positive association with stunting in this model. In contrast, in Pakistan, the interaction between intrinsic agency and age‐appropriate food intake was associated with a reduced risk of stunting [0.92 (0.86–0.99)] (Table 2 and Appendix 12).

## 4 Discussion

This multi‐country analysis demonstrates that women's empowerment, specifically social independence and instrumental agency, is associated with reduced risk of stunting in South Asia, particularly in Bangladesh, India and Pakistan. Improved HH WaSH facility was consistently associated with a lower risk of stunting across the region, with Nepal showing the strongest association. Furthermore, adherence to age‐appropriate dietary practices for children consistently correlated with lower stunting prevalence in South Asia. The combination of women's social independence and improved WaSH facility further had an association with lower risk of stunting in Bangladesh and Pakistan. However, the interaction between women's empowerment was associated with a higher risk of stunting. These findings underscore that empowerment, WaSH, and nutrition interventions interact in context‐specific ways rather than simple additive effects.

Women's social independence consistently emerged as the strongest predictor across all countries except for Nepal. Its significance likely lies in its role in delayed marriage, higher education and improved access to information, factors that enable women to make informed life choices as described in Kabeer's framework of empowerment (Kabeer 1999). Though the prevalence of child marriage in South Asia declined from 1985 to 2010, it remains concentrated in specific regions and cultural groups, contributing to low birth weight and intergenerational cycle of stunting and reflects entrenched poverty and social norms, leaving women's social independence largely stagnant over the past two decades in much of South Asia (Amin 1997; MoHP 2023; NIPORT 2024; Subramanee et al. 2022; Vir 2016). Even though, similar social structure, there was an association between social independence and stunting in Nepal, while in Bangladesh exhibits a positive association. The high rates of child marriage and school dropout which hinders women's “power within” and “power to,” reducing autonomy in decisions related to food and health in Nepal. In contrast, substantial expansion of the microcredit program in Bangladesh improves women's access autonomy of food and health (Kabeer 1999; MoHP 2023; Navarro‐Mantas et al. 2022; NIPORT 2024). Whereas positive patterns were noted in India and Pakistan. These findings highlight the potential of women's social independence in reducing childhood stunting.

Intrinsic agency, reflecting women's internal attitudes towards gender norms and violence, was independently associated with a reduced risk of stunting in Bangladesh, although the effect size was modest. This aligns with evidence that traditional gender norms and acceptance of intimate partner violence remain persistent (Rowlands 1998). However, intrinsic agency, while indicative of internal empowerment, may not translate into substantial improvements in child nutrition in the absence of enabling structural conditions. As an internalized dimension, it does not necessarily confer direct power over resources, HH decision‐making that influence child‐feeding and healthcare practices. In contrast, social independence and instrumental agency may exert more proximal effects through pathways related to resource allocation, service utilization, and caregiving behaviors. The lack of consistent associations across countries likely reflects contextual heterogeneity in how intrinsic agency operates within different sociocultural environments. The observed association in Bangladesh should, therefore, be interpreted cautiously and not generalized. Overall, these findings suggest that intrinsic agency alone is insufficient to influence stunting without concurrent improvements in other empowerment domains and broader socioeconomic conditions.

Instrumental agency, measured through women's decision‐making over large HH purchases, had a small but significant association with the risk of stunning in the pooled South Asian DHS sample, consistent with findings from 30 African countries (Yaya et al. 2020). However, this pooled association likely reflects broader regional dynamics rather than uniform progress towards achieving SDG 5 (Gender Equality). Country‐level differences in gender inequality shaped the strength and direction of this relationship with stunting. In regions with high gender inequality, low social independence, and weak instrumental agency, such as parts of South Asia, children continue to experience greater growth failure (Eom et al. 2024).

In India, instrumental agency showed both positive and negative associations with a lower risk of stunting. When adjusted for interaction between HH WaSH facility and instrumental agency, it showed an association with increased risk of stunting. In patriarchal societies, men seldom interfere with women's autonomy in matters of food and child‐care decisions; however, typically dominate large expenditures. Women's involvement in major purchases may therefore signal greater autonomy and gender equality (Huis et al. 2020). However, such women might also face constraints, such as limited time for child feeding and care, due to their additional responsibilities (Khed and Krishna 2023). In contrast, when our model was adjusted for interaction between food consumption and instrumental agency, it was associated with reduced risk of stunting independently. Low self‐efficacy and internalized gender norms among South Asian women often limit their decision‐making autonomy to domestic work and childcare; however, the indirect effect of instrumental agency for the association of reduced risk of stunting of offsprings became negative for accessing food security (Khed and Krishna 2023; Salinger et al. 2024).

We observed a strong contribution of WaSH in reducing the risk of stunting in South Asia and across countries. Improved WaSH practice lowers exposure to enteric pathogens, thereby reducing risk of diarrheal diseases and environmental enteric dysfunction, both of which can improve early childhood growth by preventing nutrient malabsorption (Reese et al. 2019).

Additionally, there is a synergistic effect between women's empowerment and HH WaSH facility with the association of decreased risk of stunting. WaSH interventions reduce fecal contamination and diarrheal disease, supporting linear growth among young children (Girma et al. 2024; Wolf et al. 2022), as seen in Pakistan where better WaSH practices are associated with lower stunting (Batool et al. 2023). Improved WaSH often indicates more advantaged HHs, including higher maternal education and later marriage, which may enhance women's autonomy in food and health decisions. However, sustained impact depends on consistent behavioral adherence, as poor implementation can undermine WaSH benefits (Pickering et al. 2019). Our findings suggest women's empowerment strengthens the effect of WaSH by enabling women to enforce hygiene practices, allocate resources, and maintain sanitary environments.

Moreover, the effect of WaSH varied across countries, underscoring contextual differences in how women's empowerment interacts with covariates. In Bangladesh, India, and Pakistan, empowerment was linked to HH WaSH facility, while in Nepal, WaSH was independent of empowerment and had the strongest positive association on lower risk of stunting. Nepal's strong government commitment and budgetary support for WaSH have contributed to improvements and reduced stunting (UNICEF 2024). In contrast, Bangladesh directed most WaSH funding to urban areas, despite 70% of the population living in rural settings, leaving *haor*, hilly, and coastal communities underserved (WaterAid & PPRC 2022). In India, underutilization of WaSH budgets and weak coordination across institutions limit effective implementation (Mukhopadhyay and Raman 2024; USAID 2020). In Pakistan, annual budget gaps persist, constraining progress (WaterAid 2020).

Age‐appropriate food consumption was significantly associated with reduced stunting risk, indicating that nutrition‐specific interventions can directly improve child growth outcomes. However, previous research has reported trade‐offs between women's empowerment and targeted nutrition strategies (Kumar et al. 2018). In South Asia, nutrition‐specific interventions (such as maternal and child nutrition programs) have shown greater impact on stunting reduction, while nutrition‐sensitive strategies (such as women's empowerment through agriculture) yielded limited benefits (Kumar et al. 2018). Similarly, evidence from Eastern Africa suggests that women's empowerment alone does not improve child nutrition, underscoring the need for integrated approaches that combine empowerment and nutrition‐sensitive interventions (Komakech et al. 2022). We observed trade‐offs between social independence and food intake, where their interaction was linked to increased stunting risk. This aligns with frameworks (Stewart et al. 2013; Smith et al. 2025) describing child feeding as a complex, context‐dependent behavior influenced by barriers such as traditional norms, limited knowledge, maternal mental health, control over resources, workload, and social support. These factors may impede optimal feeding practices, showing that a “one‐size‐fits‐all” approach is ineffective. Furthermore, trade‐offs between instrumental agency and food intake may reflect HH dynamics, food taboos, and hierarchies. In South Asia, even women with greater decision‐making autonomy may face constraints due to entrenched beliefs and senior family members' dominance (UNICEF 2021a; Navarro‐Mantas et al. 2022; Quisumbing et al. 2021). Expression of agency may also conflict with gender norms and power structures, limiting improved feeding practices.

Furthermore, South Asian context women are often burdened with farming and domestic work, reducing time for breastfeeding, food preparation, and healthcare, which affects child growth (Rao et al. 2019). Our findings also question the effectiveness of nutrition‐sensitive strategies like engaging women in agriculture to improve dietary diversity in reducing stunting in Bangladesh (Waid et al. 2024). Strong evidence supports nutrition‐specific interventions focused on maternal and child nutrition as most effective for stunting reduction in this region (Vir 2016; Bhutta et al. 2013). Overall, our results highlight the need for integrated strategies, combining nutrition‐specific and nutrition‐sensitive approaches, to effectively address stunting in South Asia.

### 4.1 Strengths and limitations

This study leverages large, nationally representative DHS datasets with standardized measures and a novel GLMM framework to account for country‐level heterogeneity. By analyzing data from four South Asian countries and focusing on age‐appropriate food intake rather than dietary diversity, this study provides unique regional insights into the intersection of women's empowerment, child feeding, and WaSH in reducing stunting.

However, the findings should be interpreted in light of their limitations. The first limitation of our study is the cross‐sectional design of the DHS data, which prevents us from making causal inferences. Second, the SWPER index, derived from DHS data, does not fully capture the multidimensional nature of women's empowerment, failing to include critical aspects such as time allocation for childcare. In addition, our sample lacks generalizability to single, divorced, or widowed women, since the SWPER only includes partnered women. Third, we did not conduct Cronbach's statistics prior to performing CFA, as we perform GSEM to measure women's empowerment measures. In addition, we used a single GSEM model for each domain of women empowerment and obtained one AIC and one BIC for each model. Since we did not build simpler models for each of the outcome indicators, we are unable to conclusively assess the goodness of fit of the GSEM models. Fourth, it was not possible to construct a comprehensive WaSH facility composite following WHO/UNICEF Joint Monitoring Prograame framework, as the DHS dataset contains only information on improved water sources and sanitation and not on actual WaSH practices (WHO and UNICEF 2025). Finally, several important determinants of stunting, including HH food security, genetic and epigenetic factors, food security, wealth index, maternal BMI, infectious diseases, and health‐seeking behaviors, could not be incorporated in the models due to data limitations and collinearity issues.

## 5 Conclusion

This study generates unique regional evidence demonstrating that domains of women's empowerment, particularly social independence, alongside HH improved WaSH facility, and age‐appropriate child feeding significantly reduce the risk of stunting among children in South Asia. Women's empowerment plays a positive direct role in mitigating stunting and reveals both synergies and tradeoffs when interacting with HH WaSH facility and age‐appropriate food consumption, respectively. Integrated strategies that combine women's empowerment, safe water and sanitation and exclusive nutrition‐specific interventions are essential to accelerate progress towards SDG 2 (zero hunger), 5 (gender equity) and 6 (clean water and sanitation).

## Author Contributions

S.P.J., R.B. and J.V.D.S. conceptualized the research question. S.P.J., R.L., R.B. and J.V.D.S. conceptualized the analysis plan. S.P.J. conducted all analyses with input from Regien Biesma, M.K.B. and J.V.D.S. S.P.J. wrote the first draft of the article. All authors contributed to critically revising the manuscript and gave final approval of the version to be published.

## Conflicts of Interest

The authors declare no conflicts of interest.

## Supporting information

Supporting File.

## Data Availability

The data that support the findings of this study are available in DHS program at https://dhsprogram.com/data/dataset_admin/index.cfm. These data were derived from the following resources available in the public domain:‐Demographic health survey, https://dhsprogram.com/data/dataset_admin/index.cfm.
