# Supplementary material for: Women's Empowerment, WaSH, and Child Feeding: Implications for Childhood Stunting in South Asia
Source: Matern Child Nutr. 2026 Jul 17;22(3):e70223. doi: 10.1111/mcn.70223 (PMC13376839; doi:10.1111/mcn.70223)
Supplement: Supplementary file 1 — Supporting File. [file MCN-22-e70223-s001.docx]

**Appendix**

**Appendix 1: Literature review for exploring potential confounders of stunting in south Asia**

***Matrix: Potential confounders of stunting among under-five children.***

| **Category** | **Potential confounder** | **Reference** |
| --- | --- | --- |
| **Social** | Intimate partner violence; Female autonomy of decision-making; social independence; access to ad control over resources; pervasive disempowerment within patriarchal structures; early marriage, dowry transactions, limited maternal education, skewed sex ratios at birth, birth order, child sex preference, child vaccination disparities, and inadequate healthcare access | Lakhdir et al., 2024; Shroff et al., 2009; Carlson et al., 2015; Quisumbing et al., 2021; Onah et al., 2021; Abdullah et al., 2023; Aguayo & Menon, 2016; Cunningham et al., 2019; Dewey, 2016; Kim et al., 2017; Zodpey & Negandhi, 2020; Wali et al., 2020 |
| **Environmental** | water, sanitation, and hygiene (WaSH) practices; rural residence | Abdullah et al., 2023; Kim et al., 2017; Soofi et al., 2023; Rah et al., 2015; Rahman et al., 2023 |
| **Nutritional** | Maternal BMI; Maternal stature; food security, duration of breastfeeding, complementary feeding minimum dietary diversity; appropriate meal frequency | Abdullah et al., 2023; Kim et al., 2017; Victora et al., 2010 |
| **Health** | Diarrhoea, ARI | Girma et al., 2024; Wolf et al., 2022 |

In South Asia, stunting among children under five is shaped by a complex interplay of social, environmental, and nutritional determinants. Empirical evidence underscores that restricted maternal autonomy in accessing financial resources and markets, coupled with exposure to intimate partner violence (IPV), substantially elevates the risk of stunting in children (Lakhdir et al., 2024; Shroff et al., 2009). Additionally, research suggests that the association between women's empowerment and childhood stunting is comparatively weaker in South Asia, primarily due to limited female autonomy and the high prevalence of IPV (Carlson et al., 2015; Quisumbing et al., 2021). Conversely, greater social independence and decision-making autonomy among women are linked to significant reductions in childhood stunting (Onah et al., 2021). The low social status of women in South Asia—exacerbated by restricted access to and control over resources, pervasive disempowerment within patriarchal structures, and socio-cultural practices such as early marriage, dowry transactions, limited maternal education, skewed sex ratios at birth, birth order, child sex preference, child vaccination disparities, and inadequate healthcare access—substantially contributes to stunting among offspring (Abdullah et al., 2023; Aguayo & Menon, 2016; Cunningham et al., 2019; Dewey, 2016; Kim et al., 2017; Zodpey & Negandhi, 2020; Wali et al., 2020). This risk is further exacerbated by environmental factors, including rural residence and suboptimal water, sanitation, and hygiene (WaSH) practices (Abdullah et al., 2023; Kim et al., 2017; Soofi et al., 2023; Rah et al., 2015; Rahman et al., 2023). Moreover, maternal anthropometric indicators play a critical role in mitigating childhood stunting. Research demonstrates that a higher maternal body mass index (BMI) and greater maternal stature serve as protective factors against intrauterine growth restriction, consequently, reduces incidence of stunting. The first 1,000 days—from conception to a child’s second birthday—constitute a critical period for stunting prevention through enhanced food security, including duration of breastfeeding, adequate complementary feeding, minimum dietary diversity, and appropriate meal frequency (Abdullah et al., 2023; Kim et al., 2017; Victora et al., 2010).

**Reference:**

Abdulla, F., Rahman, A., & Hossain, M. M. (2023). Prevalence and risk predictors of childhood stunting in Bangladesh. PloS one, 18(1), e0279901. <https://doi.org/10.1371/journal.pone.0279901>

Aguayo, V. M., & Menon, P. (2016). Stop stunting: improving child feeding, women's nutrition and household sanitation in South Asia. Maternal & child nutrition, 12 Suppl 1(Suppl 1), 3–11. <https://doi.org/10.1111/mcn.12283>

Carlson GJ, Kordas K, Murray-Kolb LE. Associations between women's autonomy and child nutritional status: a review of the literature. Matern Child Nutr. 2015 Oct;11(4):452-82. doi: 10.1111/mcn.12113. Epub 2014 Feb 13. PMID: 24521434; PMCID: PMC6860340.

Cunningham K, Ferguson E, Ruel M, Uauy R, Kadiyala S, Menon P, Ploubidis G. Water, sanitation, and hygiene practices mediate the association between women's empowerment and child length-for-age z-scores in Nepal. Matern Child Nutr. 2019 Jan;15(1):e12638. doi: 10.1111/mcn.12638. Epub 2018 Jul 26. PMID: 30047247; PMCID: PMC7198941.

Dewey K. G. (2016). Reducing stunting by improving maternal, infant and young child nutrition in regions such as South Asia: evidence, challenges and opportunities. Maternal & child nutrition, 12 Suppl 1(Suppl 1), 27–38. <https://doi.org/10.1111/mcn.12282>

Kim, R., Mejía-Guevara, I., Corsi, D. J., Aguayo, V. M., & Subramanian, S. V. (2017). Relative importance of 13 correlates of child stunting in South Asia: Insights from nationally representative data from Afghanistan, Bangladesh, India, Nepal, and Pakistan. Social science & medicine (1982), 187, 144–154. https://doi.org/10.1016/j.socscimed.2017.06.017

Lakhdir MPA, Ambreen S, Sameen S, Asim M, Batool S, Azam I, Usmani BA, Iqbal R. Association between maternal experiences of intimate partner violence and child stunting: a secondary analysis of the Demographic Health Surveys of four South Asian countries. BMJ Open. 2024 Jan 19;14(1):e071882. doi: 10.1136/bmjopen-2023-071882. PMID: 38245010; PMCID: PMC10806751.

Onah MN. Women's empowerment and child nutrition in South-Central Asia; how important is socioeconomic status? SSM Popul Health. 2020 Dec 13;13:100718. doi: 10.1016/j.ssmph.2020.100718. PMID: 33426264; PMCID: PMC7779324.

Rah JH, Cronin AA, Badgaiyan B, Aguayo VM, Coates S, Ahmed S. Household sanitation and personal hygiene practices are associated with child stunting in rural India: a cross-sectional analysis of surveys. BMJ Open. 2015 Feb 12;5(2):e005180. doi: 10.1136/bmjopen-2014-005180. PMID: 25678539; PMCID: PMC4330332.

Rahman MT, Jahangir Alam M, Ahmed N, Roy DC, Sultana P. Trend of risk and correlates of under-five child undernutrition in Bangladesh: an analysis based on Bangladesh Demographic and Health Survey data, 2007-2017/2018. BMJ Open. 2023 Jun 12;13(6):e070480. doi: 10.1136/bmjopen-2022-070480. PMID: 37308267; PMCID: PMC10277110.

Shroff M, Griffiths P, Adair L, Suchindran C, & Bentley M. Maternal autonomy is inversely related to child stunting in Andhra Pradesh, India. Maternal & child nutrition. 2009. 5(1), 64–74. https://doi.org/10.1111/j.1740-8709.2008.00161.x

Soofi SB, Khan A, Kureishy S, Hussain I, Habib MA, Umer M, Ariff S, Sajid M, Rizvi A, Ahmed I, Iqbal J, Ahmed KM, Achakzai ABK, Bhutta ZA. Determinants of Stunting among Children under Five in Pakistan. Nutrients. 2023;15(15):3480. doi: 10.3390/nu15153480. PMID: 37571417; PMCID: PMC10421501.

Victora CG, de Onis M, Hallal PC, Blössner M, Shrimpton R. Worldwide timing of growth faltering: revisiting implications for interventions. Pediatrics. 2010 Mar;125(3):e473-80. doi: 10.1542/peds.2009-1519. Epub 2010 Feb 15. PMID: 20156903.

Wali N, Agho KE, Renzaho AMN. Factors Associated with Stunting among Children under 5 Years in Five South Asian Countries (2014-2018): Analysis of Demographic Health Surveys. Nutrients. 2020 Dec 18;12(12):3875. doi: 10.3390/nu12123875. PMID: 33352949; PMCID: PMC7767090.

Zodpey S, Negandhi P. Inequality in health and social status for women in India - A long-standing bane. Indian J Public Health. 2020 Oct-Dec;64(4):325-327. doi: 10.4103/ijph.IJPH_1312_20. PMID: 33318379.

**Appendix 2**

**Directed Acyclic Graph (DAG)**

To further illustrate the conceptual framework underlying covariate selection, we developed a Directed Acyclic Graph (DAG) based on the existing literature on determinants of childhood stunting in South Asia. The DAG depicts the hypothesized relationships among women’s empowerment (SWPER-global domains), mediators (child feeding practices and WaSH), socioeconomic and maternal characteristics, and childhood stunting. The DAG was used to identify a minimal sufficient adjustment set for estimating the direct effect of the exposure variables on stunting.

**Supplementary Figure:** Directed acyclic graph illustrating the hypothesized relationships between women’s empowerment, WaSH, child feeding practices, and childhood stunting used to identify adjustment variables


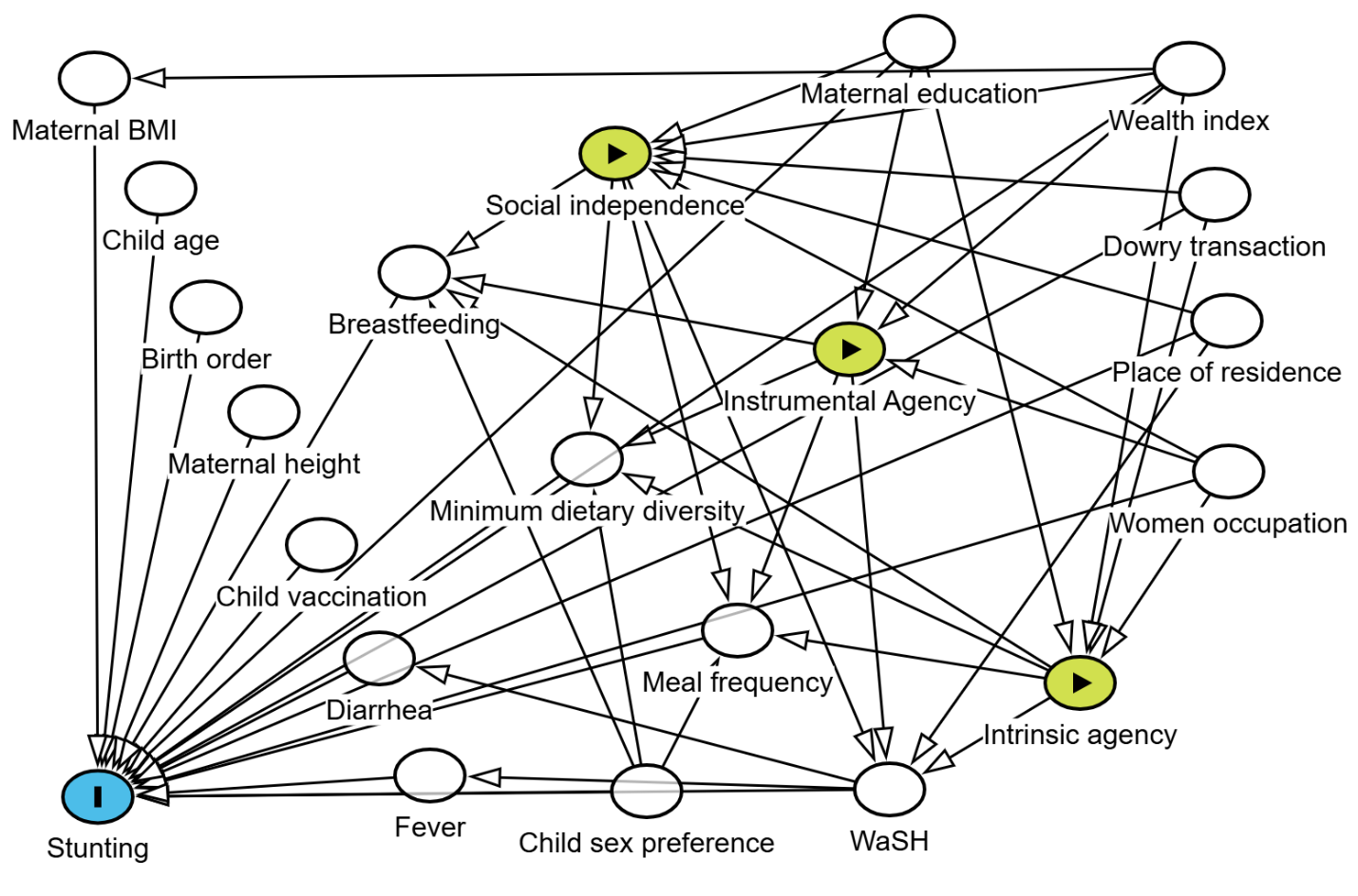


**Appendix 3: List of covariates and study variables, with definitions and coding schemes used for generating dummy variables**

| **Covariates** | **Variable/s used in DHS data sets** | **Definition utilized** | **Coding for generating binary variables** |
| --- | --- | --- | --- |
| **Social factors** |  |  |  |
| Wealth index | V190 | We utilized the existing wealth index variables from the data sets | A total of four variables were derived from the wealth index variable, representing wealth quintiles 2, 3, 4, and 5. Each of these quintiles was coded as “1” for the corresponding variable, while wealth quintile 1 was designated as the reference category and coded as “0.” |
| Employment of women | V717 | Women employed in professional, clerical, sales and service, skilled manual, unskilled manual, domestic services, or agricultural occupations were classified as participating in income-generating activities. In contrast, those not engaged in any occupation were categorized as not having an income. | Women engaged in income-generating activities were coded as “1,” while those not involved in income-generating activities were coded as “0.” |
| Age group of the children | HW1 | The age of children (HW1) was categorized into three groups based on recommended feeding practices:   - **0–5 months:** Exclusive breastfeeding is recommended during this period. - **6–23 months:** Continued breastfeeding along with a minimum acceptable diet is recommended. - **24–59 months:** A minimum dietary diversity is recommended in their daily diet. | Three variables were created based on the children's age groups. For each specific age group, children within that category were coded as “1,” while those in other age groups were coded as “0.” |
| Sex of the children | B4 | The sex of the children was already recorded in the DHS dataset. | Female children were coded as “1,” while male children were coded as “0.” |
| **Environmental factors** |  |  |  |
| Palce of residence | V102 | The place of residence was categorized into two groups: rural and urban. | Rural areas were coded as “0,” while urban areas were coded as “1.” |
| Use of sanitary latrine | V116 | Toilet facilities, including flush to pipe, flush to septic tank, flush to pit latrine, basic flush, ventilated improved pit latrine, pit latrine with slab, and composting toilets, were categorized as having a sanitary latrine in the household (Croft et al., 2023). | A sanitary latrine was coded as “1,” while non-sanitary latrines were coded as “0.” |
| Use of improved sources of water | V113 | Sources such as piped into dwelling, piped to yard/plot, public tap/standpipe, piped to neighbor, tube well or borehole, protected well, protected spring, rainwater, tanker truck, cart with small tank, and bottled water were classified as improved water sources for household members (Croft et al., 2023). | Improved sources of water were coded as “1,” while other sources were coded as “0.” |
| Household WaSH facility | V113 & V116 | The "WaSH" facility was a composite variable derived from the variables for sanitary latrine and improved sources of water. A household that had both a sanitary latrine and improved sources of water was considered to have a WaSH facility, while households without both were classified as not having a WaSH facility. Toilet facilities, including flush to pipe, flush to septic tank, flush to pit latrine, basic flush, ventilated improved pit latrine, pit latrine with slab, and composting toilets, were categorized as having a sanitary latrine in the household (Croft et al., 2023).Water sources such as piped into dwelling, piped to yard/plot, public tap/standpipe, piped to neighbor, tube well or borehole, protected well, protected spring, rainwater, tanker truck, cart with small tank, and bottled water were classified as improved water sources for household members (Croft et al., 2023). | Households with a WaSH facility were coded as “1,” while those without a WaSH facility were coded as “0.” |
| **Nutritional factors** |  |  |  |
| Maternal stature | V438 | Before merging the datasets, the height of the women was converted into centimeters (cm). After merging the datasets, height was categorized into four groups to assess the effect of maternal short stature and child stunting. The categories for women’s stature were:   - <145.0 cm - 145.0–149.9 cm - 150.0–154.9 cm - ≥155.0 cm   These categories were adapted from an earlier study by Ozaltin et al. (2010). | Three variables were generated for the height categories of women. For each specific group, women within that height range were coded as “1,” while those outside the range were coded as “0.” A height of "<145.0 cm" was considered the reference category and was coded as "0." |
| Maternal BMI | V445 | The data were categorized using the classification provided by the WHO (Stevens et al.,2000). The BMI categories are as follows:   - Underweight: BMI < 18.50 kg/m² - Normal weight: BMI ≥ 18.50 to 24.9 kg/m² - Overweight: BMI ≥ 25.00 to 29.9 kg/m² - Obesity: BMI ≥ 30.00 kg/m² | Three variables were generated for the BMI categories of women. For each specific group, women within that BMI range were coded as "1," while those outside the range were coded as "0." A BMI of <18.5 kg/m² was always considered the reference category and coded as "0." |
| Underweight of the children | HW71 | A child was considered underweight if the Weight-for-Age Z (WAZ) score was less than -2 standard deviations (SD) from the median of the WHO Child Growth Standards reference population (WHO, 2006). | Underweight children were coded as “1,” while those who were not underweight were coded as “0.” |
| Wasting of the children | HW72 | A child was considered to be suffering from wasting if the Weight-for-Height Z (WHZ) score was less than -2 standard deviations (SD) from the median of the WHO Child Growth Standards reference population (WHO, 2006). | Wasted children were coded as “1,” while those who were not wasted were coded as “0.” |
| Age-appropriate food intake | HW1  V404  V409  V410  V414S  M39  V414E  V414F  V414O  V414  V414P  V414H  V414M  V414N  V414G  V414I  V414J  V414K V414L | A composite variable for age-appropriate food intake among children under five was created and categorized into two groups based on adherence to exclusive breastfeeding (EBF), meal frequency, and dietary diversity, as per age-specific guidelines during the 24 hours preceding the interview (NIPORT, 2022). Dietary intake recommendations for children under five are tailored to their specific age groups, and this study followed the dietary guidelines outlined in the BDHS report (NIPORT, 2022). For infants aged 0–5 months, exclusive breastfeeding is the recommended and ideal source of nutrition. Breastfed infants aged 6–8 months should receive two meals per day, with each meal including foods from at least four of the seven food groups. Breastfed children aged 9–23 months are advised to have three meals per day, ensuring the inclusion of foods from at least four of the seven food groups. Non-breastfed children aged 6–23 months should consume three meals daily, with their diet including foods from at least four food groups, including dairy. For children aged 24–59 months, the dietary recommendation is to consume food from at least four food groups within the preceding 24 hours. These guidelines ensure that children’s dietary needs are met based on their developmental stage and nutritional requirements (NIPORT, 2024). Children who had an age-appropriate diet in the last 24 hours were coded as “1,” while those who did not were coded as “0.” | Children who had an age-appropriate diet in the last 24 hours were coded as “1,” while those who did not were coded as “0.” |
| **Health factors** |  |  |  |
| Suffering from diarrhoea | H11 | Children who had experienced diarrhea in the two weeks preceding the interview were classified as having had diarrhea. | Children who had diarrhea were coded as “1,” while those who did not were coded as “0.” |
| Suffering from fever | H22 | Children who had experienced fever in the two weeks preceding the interview were classified as having had a fever. | Children who had fever was coded as “1”, otherwise “0” |
| Suffering from ARI | H31B, H31c | Children who exhibited symptoms of Acute Respiratory Infection (ARI), such as breathing difficulties and rapid breathing, or both, in the two weeks preceding the interview were classified as having had ARI. | Children who exhibited symptoms of ARI were coded as “1,” while those who did not were coded as “0.” |

**Reference:**

Croft, Trevor N., Allen, Courtney K., Zachary, Blake W., et al. 2023. Guide to DHS Statistics. Rockville, Maryland, USA: ICF.”

World Health Organization (WHO) and the United Nations Children’s Fund (UNICEF). 2021.Indicators for assessing infant and young child feeding practices: definitions and measurement methods. Geneva: Licence: CC BYNC-SA 3.0 IGO; <https://creativecommons.org/licenses/by-nc-sa/3.0/igo>.

National Institute of Population Research and Training (NIPORT) and ICF. (2024). Bangladesh Demographic and Health Survey 2022: Key Indicators Report. Dhaka, Bangladesh, and Rockville, Maryland, USA: NIPORT and ICF.

WHO, M. (2006). Growth, Reference, Study, Group. *WHO Child Growth Standards: Length/height-for-age, weight-for-age, weight-for-length, weight-for-height and body mass index-for-age: Methods and development. Geneva: World Health Organization*, *312*. <https://www.who.int/publications/i/item/924154693X>

**Appendix 4: Frequency distribution of HAZ by the different covariates in South Asian countries (weighted)**

| **Covariates** | **Pooled** | | **Bangladesh** | | **India** | | **Nepal** | | **Pakistan** | |
| --- | --- | --- | --- | --- | --- | --- | --- | --- | --- | --- |
|  | **n=37620** | | **n=6271** | | **n=26398** | | **n=1539** | | **n=3412** | |
|  | **HAZ<-2.00 SD** | | **HAZ<-2.00 SD** | | **HAZ<-2.00 SD** | | **HAZ<-2.00 SD** | | **HAZ<-2.00 SD** | |
|  | **n=13207** | | **n=2015** | | **n=9412** |  | **n=434** |  | **n=1346** |  |
|  | **n** | **%** | **n** | **%** | **n** | **%** | **n** | **%** | **n** | **%** |
| **Maternal covariates** |  |  |  |  |  |  |  |  |  |  |
| Maternal height in cm |  |  |  |  |  |  |  |  |  |  |
| ≥155.0 | 2779 | 24.45 | 240 | 17.11 | 1948 | 24.91 | 75 | 16.52 | 516 | 39.57 |
| 154.9-150.0 | 4182 | 33.35 | 585 | 27.26 | 2988 | 33.94 | 140 | 26.17 | 469 | 44.45 |
| 149.9-145.0 | 4033 | 41.93 | 719 | 38.68 | 2884 | 42.36 | 153 | 36.78 | 277 | 51.78 |
| <145.0 | 2213 | 54.03 | 471 | 54.58 | 1892 | 53.69 | 66 | 49.25 | 84 | 62.69 |
| Maternal BMI in kg/m^2^ |  |  |  |  |  |  |  |  |  |  |
| Obesity ≥30.0 | 600 | 27.27 | 70 | 20.83 | 331 | 27.49 | 15 | 23.08 | 184 | 30.92 |
| Overweight ≥25.0- 29.9 | 2458 | 28.34 | 434 | 25.32 | 1497 | 28.14 | 85 | 21.68 | 442 | 35.39 |
| Normal 18.5-24.9 | 7346 | 36.33 | 1093 | 33.48 | 5416 | 36.57 | 259 | 29.67 | 578 | 45.40 |
| Underweight <18.5 | 2803 | 42.95 | 418 | 43.72 | 2168 | 42.80 | 75 | 35.89 | 142 | 48.14 |
| Women involve in income |  |  |  |  |  |  |  |  |  |  |
| Yes | 4092 | 36.64 | 980 | 34.53 | 2597 | 38.03 | 330 | 30.03 | 204 | 45.03 |
| No | 9100 | 34.43 | 1035 | 30.15 | 6819 | 34.79 | 104 | 23.64 | 1142 | 38.59 |
| **Child covariates** |  |  |  |  |  |  |  |  |  |  |
| Child age category in month |  |  |  |  |  |  |  |  |  |  |
| 0-5 months | 782 | 22.25 | 138 | 19.06 | 552 | 24.24 | 28 | 17.72 | 64 | 18.03 |
| 6-23 months | 3725 | 34.11 | 599 | 31.59 | 2701 | 35.54 | 108 | 24.83 | 317 | 32.05 |
| 24-59 months | 8700 | 37.52 | 1278 | 35.00 | 6159 | 37.28 | 298 | 31.50 | 965 | 46.66 |
| Sex of the children |  |  |  |  |  |  |  |  |  |  |
| Male | 7054 | 36.30 | 1065 | 32.57 | 5070 | 37.19 | 232 | 28.68 | 687 | 39.92 |
| Female | 6153 | 33.83 | 950 | 31.66 | 4342 | 34.01 | 202 | 27.67 | 659 | 38.97 |
| Age-appropriate diet intake during last 24 hours |  |  |  |  |  |  |  |  |  |  |
| Yes | 1346 | 29.07 | 278 | 24.91 | 940 | 31.88 | 39 | 23.49 | 89 | 22.31 |
| No | 11861 | 35.95 | 1737 | 33.70 | 8472 | 36.13 | 395 | 28.77 | 1257 | 41.72 |
| Suffered from diarrhoea during the last two weeks |  |  |  |  |  |  |  |  |  |  |
| Yes | 1135 | 37.83 | 88 | 28.57 | 731 | 39.41 | 42 | 24.00 | 274 | 41.33 |
| No | 12072 | 34.87 | 1927 | 32.32 | 8681 | 35.37 | 392 | 28.74 | 1072 | 39.00 |
| Suffered from fever during last two weeks |  |  |  |  |  |  |  |  |  |  |
| Yes | 2550 | 36.28 | 684 | 33.17 | 1241 | 37.61 | 97 | 25.59 | 528 | 41.03 |
| No | 10657 | 34.84 | 1331 | 31.62 | 8171 | 35.38 | 337 | 29.05 | 818 | 38.49 |
| Suffered from AIR during last two weeks |  |  |  |  |  |  |  |  |  |  |
| Yes | 1174 | 36.96 | 264 | 33.21 | 610 | 37.63 | 25 | 21.37 | 275 | 42.77 |
| No | 12033 | 34.93 | 1751 | 31.98 | 8802 | 35.52 | 409 | 28.76 | 1071 | 38.68 |
| WAZ |  |  |  |  |  |  |  |  |  |  |
| Non-underweight | 6006 | 21.94 | 918 | 19.03 | 4164 | 22.29 | 224 | 18.04 | 700 | 26.63 |
| Underweight | 7201 | 70.32 | 1097 | 76.81 | 5248 | 68.03 | 210 | 70.71 | 646 | 82.50 |
| WHZ |  |  |  |  |  |  |  |  |  |  |
| Non-wasting | 11617 | 36.55 | 1824 | 31.86 | 8153 | 37.92 | 392 | 27.47 | 1248 | 39.87 |
| Wasting | 1590 | 27.24 | 191 | 34.98 | 1259 | 25.71 | 42 | 37.50 | 98 | 34.75 |
| **Household covariates** |  |  |  |  |  |  |  |  |  |  |
| Wealth index |  |  |  |  |  |  |  |  |  |  |
| Richest | 1195 | 21.45 | 201 | 16.83 | 837 | 23.39 | 29 | 15.26 | 128 | 21.05 |
| Fourth | 1895 | 28.00 | 348 | 28.76 | 1324 | 28.07 | 41 | 16.94 | 182 | 30.38 |
| Middle | 2429 | 33.68 | 342 | 31.84 | 1770 | 34.14 | 76 | 24.92 | 241 | 37.19 |
| Poorer | 3327 | 38.84 | 509 | 39.55 | 2373 | 38.65 | 84 | 27.63 | 361 | 43.29 |
| Poorest | 4361 | 45.88 | 615 | 49.84 | 3108 | 45.85 | 204 | 40.96 | 434 | 60.45 |
| Residence |  |  |  |  |  |  |  |  |  |  |
| Urban | 3066 | 29.64 | 612 | 26.92 | 1715 | 30.42 | 183 | 23.02 | 556 | 33.90 |
| Rural | 10141 | 37.18 | 1403 | 35.09 | 7697 | 37.07 | 251 | 33.74 | 790 | 44.58 |
| Used sanitary latrine |  |  |  |  |  |  |  |  |  |  |
| Yes | 8798 | 32.48 | 1028 | 28.87 | 6483 | 33.17 | 346 | 26.31 | 941 | 35.26 |
| No | 4409 | 41.87 | 987 | 36.42 | 2929 | 42.75 | 88 | 39.29 | 405 | 54.51 |
| Used improved sources of water |  |  |  |  |  |  |  |  |  |  |
| Yes | 12380 | 34.97 | 1969 | 31.94 | 8774 | 35.69 | 421 | 28.09 | 1216 | 38.52 |
| No | 827 | 37.35 | 46 | 43.40 | 638 | 35.19 | 13 | 32.50 | 130 | 50.98 |
| Had water and sanitation practice |  |  |  |  |  |  |  |  |  |  |
| Yes | 8297 | 32.36 | 1005 | 28.66 | 6082 | 33.18 | 336 | 26.25 | 874 | 34.61 |
| No | 4910 | 41.00 | 1010 | 36.54 | 3330 | 41.28 | 98 | 37.84 | 472 | 53.21 |
| Country |  |  |  |  |  |  |  |  |  |  |
| India | 9412 | 35.65 | - | - | - | - | - | - | - | - |
| Bangladesh | 2015 | 32.13 | - | - | - | - | - | - | - | - |
| Nepal | 434 | 28.20 | - | - | - | - | - | - | - | - |
| Pakistan | 1346 | 39.45 | - | - | - | - | - | - | - | - |

HAZ- Height-for-age Z score ; WAZ- Weight-for-age Z score ; WHZ- Weight-for-height Z score

**Appendix 5: Binary association between under-five children stunting and covariates (weighted)**

| **Covariates** | **Pooled** | | **Bangladesh** | | **India** | | **Nepal** | | **Pakistan** | |
| --- | --- | --- | --- | --- | --- | --- | --- | --- | --- | --- |
|  | **Unadjusted RR** | **95% CI** | **Unadjusted RR** | **95% CI** | **Unadjusted RR** | **95% CI** | **Unadjusted RR** | **95% CI** | **Unadjusted RR** | **95% CI** |
| **SWPER** |  |  |  |  |  |  |  |  |  |  |
| Social Independence | 0.77*** | 0.74, 0.80 | 0.70*** | 0.63, 0.78 | 0.75*** | 0.71, 0.78 | 0.72*** | 0.58, 0.90 | 0.62*** | 0.55, 0.70 |
| Intrinsic Agency | 0.98*** | 0.07, 0.99 | 0.96*** | 0.94, 0.98 | 0.99 | 0.98, 1.00 | 0.95** | 0.91, 0.99 | 0.96*** | 0.94, 0.97 |
| Instrumental Agency | 0.98*** | 0.07, 0.99 | 0.99 | 0.97, 1.02 | 0.99 | 0.97, 1.00 | 0.96 | 0.92, 1.00 | 0.95*** | 0.92, 0.98 |
|  |  |  |  |  |  |  |  |  |  |  |
| **Maternal covariates** |  |  |  |  |  |  |  |  |  |  |
| Maternal height in cm |  |  |  |  |  |  |  |  |  |  |
| ≥155.0 (=1) | 1 |  | 1 |  | 1 |  | 1 |  | 1 |  |
| 154.9-150.0 (=1) | 0.60*** | 0.57, 0.63 | 0.45*** | 0.39, 0.52 | 0.61*** | 0.57, 0.64 | 0.47*** | 0.36, 0.62 | 0.62*** | 0.54, 0.71 |
| 149.9-145.0 (=1) | 0.89*** | 0.86, 0.93 | 0.78*** | 0.71, 0.86 | 0.90*** | 0.86, 0.95 | 0.88* | 0.82. 1.08 | 1.08* | 0.95, 1.23 |
| <145.0 (=1) | 1.31*** | 1.26, 1.36 | 1.38*** | 1.20, 1.42 | 1.31*** | 1.25, 1.37 | 1.44*** | 1.19, 1.75 | 1.51*** | 1.32, 1.72 |
| Maternal BMI in kg/m^2^ |  |  |  |  |  |  |  |  |  |  |
| Obesity ≥30.0 (=0) | 1 |  | 1 |  | 1 |  | 1 |  | 1 |  |
| Overweight ≥25.0- 29.9 (=1) | 0.76*** | 0.72, 0.80 | 0.76*** | 0.68, 0.84 | 0.76*** | 0.71, 0.82 | 0.64*** | 0.50, 0.82 | 0.77*** | 0.67, 0.88 |
| Normal 18.5-24.9 (=1) | 1.08*** | 1.04, 1.12 | 1.04* | 0.96, 1.12 | 1.06*** | 1.02, 1.11 | 1.17* | 0.96, 1.41 | 1.32** | 1.17, 1.49 |
| Underweight <18.5(=1) | 1.28*** | 1.23, 1.34 | 1.47*** | 1.33, 1.61 | 1.24*** | 1.18, 1.31 | 1.49*** | 1.18, 1.87 | 1.23** | 1.03, 1.46 |
| Employed women |  |  |  |  |  |  |  |  |  |  |
| Yes (=0) | 1 |  | 1 |  | 1 |  | 1 |  | 1 |  |
| No (=1) | 1.06*** | 1.01, 1.10 | 1.17*** | 1.07, 1.26 | 1.08*** | 1.03, 1.14 | 1.23* | 0.99, 1.53 | 1.28*** | 1.10, 1.48 |
|  |  |  |  |  |  |  |  |  |  |  |
| **Child covariates** |  |  |  |  |  |  |  |  |  |  |
| Child age category in month |  |  |  |  |  |  |  |  |  |  |
| 0-5 months (=0) | 1 |  | 1 |  | 1 |  | 1 |  | 1 |  |
| 6-23 months (=1) | 0.95** | 0.91, 0.99 | 0.95 | 0.86, 1.03 | 0.98 | 0.93, 1.03 | 0.86 | 0.69, 1.07 | 0.74*** | 0.64, 0.86 |
| 24-59 months (=1) | 1.20*** | 1.15, 1.25 | 1.28*** | 1.17, 1.39 | 1.13 | 1.08, 1.19 | 1.29** | 1.05, 1.59 | 1.64*** | 1.42, 1.89 |
| Sex of the children |  |  |  |  |  |  |  |  |  |  |
| Male (=0) | 1 |  | 1 |  | 1 |  | 1 |  | 1 |  |
| Female (=1) | 0.95** | 0.92, 0.99 | 0.96 | 0.89, 1.05 | 0.94** | 0.90, 0.98 | 1.07 | 0.88, 1.29 | 0.98 | 0.87, 1.11 |
| Age-appropriate diet intake during last 24 hours |  |  |  |  |  |  |  |  |  |  |
| Yes (=0) | 1 |  | 1 |  | 1 |  | 1 |  | 1 |  |
| No (=1) | 0.78*** | 0.74, 0.83 | 0.72*** | 0.64. 0.81 | 0.84*** | 0.78, 0.91 | 0.92 | 0.66, 1.29 | 0.52*** | 0.39, 0.70 |
| Suffered from diarrhoea during the last two weeks |  |  |  |  |  |  |  |  |  |  |
| Yes (=0) | 1 |  | 1 |  | 1 |  | 1 |  | 1 |  |
| No (=1) | 0.91*** | 0.85, 0.97 | 1.11 | 0.91, 1.36 | 0.89*** | 0.82, 0.96 | 1.27 | 0.91, 1.78 | 0.88 | 0.76, 1.02 |
| Suffered from fever during last two weeks |  |  |  |  |  |  |  |  |  |  |
| Yes (=0) | 1 |  | 1 |  | 1 |  | 1 |  | 1 |  |
| No (=1) | 0.95* | 0.91, 1.00 | 0.94 | 0.87, 1.03 | 0.94* | 0.88, 1.00 | 1.11 | 0.83, 1.39 | 0.90 | 0.79, 1.02 |
| Suffered from ARI during last two weeks |  |  |  |  |  |  |  |  |  |  |
| Yes (=0) | 1 |  | 1 |  | 1 |  | 1 |  | 1 |  |
| No (=1) | 0.94 | 0.89, 1.01 | 0.97 | 0.86, 1.09 | 0.94 | 0.86, 1.03 | 1.44 | 0.92, 2.26 | 0.88 | 0.76, 1.02 |
| WAZ |  |  |  |  |  |  |  |  |  |  |
| Non-underweight (=0) | 1 |  | 1 |  | 1 |  | 1 |  | 1 |  |
| Underweight (=1) | 3.33*** | 3.22, 3.45 | 4.02*** | 0.37, 4.33 | 3.16*** | 3.02, 3.30 | 4.49*** | 3.81, 5.31 | 3.34*** | 3.00, 3.71 |
| WHZ |  |  |  |  |  |  |  |  |  |  |
| Non-wasting (=0) | 1 |  | 1 |  | 1 |  | 1 |  | 1 |  |
| Wasting (=1) | 0.80*** | 0.76, 0.85 | 1.10 | 0.96, 1.26 | 0.73*** | 0.68, 0.78 | 1.53*** | 1.15, 2.02 | 1.07 | 0.86, 1.33 |
|  |  |  |  |  |  |  |  |  |  |  |
| **Household covariates** |  |  |  |  |  |  |  |  |  |  |
| Wealth index |  |  |  |  |  |  |  |  |  |  |
| Richest (=0) | 1 |  | 1 |  | 1 |  | 1 |  | 1 |  |
| Fourth (=1) | 0.76*** | 0.72, 0.81 | 0.86*** | 0.77, 0.96 | 0.75*** | 0.70, 0.81 | 0.60*** | 0.44, 0.83 | 0.74*** | 0.62,0.89 |
| Middle (=1) | 0.94** | 0.90, 0.99 | 0.95 | 0.86, 1.07 | 0.96 | 0.90, 1.02 | 0.91 | 0.72, 1.16 | 0.79** | 0.67, 0.95 |
| Poorer (=1) | 1.18*** | 1.14, 1.23 | 1.28*** | 1.17,1.40 | 1.15*** | 1.10, 1.21 | 1.16 | 0.93, 1.45 | 1.25*** | 1.09, 1.43 |
| Poorest (=1) | 1.51*** | 1.45, 1.56 | 1.39*** | 1.28, 1.51 | 1.49*** | 1.43, 1.56 | 2.01*** | 1.69, 2.40 | 1.68*** | 1.49,1.89 |
| Residence |  |  |  |  |  |  |  |  |  |  |
| Urban (=0) | 1 |  | 1 |  | 1 |  | 1 |  | 1 |  |
| Rural (=1) | 1.31*** | 1.25, 1.37 | 1.32*** | 1.20, 1.45 | 1.27*** | 1.19, 1.35 | 1.58*** | 1.32, 1.89 | 1.30*** | 1.14, 1.49 |
| Used improved sanitary latrine |  |  |  |  |  |  |  |  |  |  |
| Yes (=0) | 1 |  | 1 |  | 1 |  | 1 |  | 1 |  |
| No | 1.30*** | 1.25, 1.35 | 1.21*** | 1.11, 1.31 | 1.31*** | 1.25, 1.37 | 1.74*** | 1.42, 2.14 | 1.55*** | 1.37, 1.74 |
| Used improved sources of water |  |  |  |  |  |  |  |  |  |  |
| Yes (=0) | 1 |  | 1 |  | 1 |  | 1 |  | 1 |  |
| No (=1) | 1.06 | 0.97, 1.15 | 1.30** | 1.02,1.67 | 0.95 | 0.86, 1.05 | 1.22 | 0.73, 2.04 | 1.50*** | 1.25, 1.80 |
| Had WASH practice |  |  |  |  |  |  |  |  |  |  |
| Yes (=0) | 1 |  | 1 |  | 1 |  | 1 |  | 1 |  |
| No (=1) | 1.29*** | 1.24, 1.34 | 1.22*** | 1.12, 1.32 | 1.27*** | 1.22, 1.33 | 1.7*** | 1.38,2.08 | 1.64*** | 1.42,1.80 |
| Country |  |  |  |  |  |  |  |  |  |  |
| India (=0) | 1 |  |  |  |  |  |  |  |  |  |
| Bangladesh (=1) | 0.88*** | 0.84, 0.92 | - | - | - | - | - | - | - | - |
| Nepal (=1) | 0.72*** | 0.65, 0.79 | - | - | - | - | - | - | - | - |
| Pakistan (=1) | 1.14*** | 1.07, 1.22 | - | - | - | - | - | - | - | - |

**** p<.01, ** p<.05, * p<.1*

*RR- relative risk; RR<1 positive association; RR=1 no association; RR>1 negative association*

*CI - Confidence Interval*

**Appendix 6: Association between Stunting among under-five children and women’s empowerment domains and all covariates (weighted)**

| **Covariates** | **Pooled** | | **Countries** | | | | | | | |
| --- | --- | --- | --- | --- | --- | --- | --- | --- | --- | --- |
|  |  |  | **Bangladesh** | | **India** | | **Nepal** | | **Pakistan** | |
|  | **adj-RR** | **95%CI** | **adj-RR** | **95%CI** | **adj-RR** | **95%CI** | **adj-RR** | **95%CI** | **Adj-RR** | **95%CI** |
| Social independence | 0.95 | 0.92-0.98 | 0.88 | 0.80-0.98 | 0.93 | 0.89-0.97 | 1.08 | 0.90-1.30 | 0.79 | 0.71-0.88 |
| Intrinsic agency | 0.99 | 0.98-0.99 | 0.98 | 0.97-1.00 | 1.00 | 0.99-1.06 | 0.97 | 0.94-1.01 | 1.002 | 0.98-1.01 |
| Instrumental agency | 0.98 | 0.97-0.99 | 0.99 | 0.97-1.01 | 0.99 | 0.97-1.16 | 0.99 | 0.95-1.03 | 0.99 | 0.96-1.02 |
| WaSH practice |  |  |  |  |  |  |  |  |  |  |
| No (=0) | 1 |  | 1 |  | 1 |  | 1 |  | 1 |  |
| Yes (=1) | 0.98 | 0.98-1.10 | 0.99 | 0.92-1.07 | 0.99 | 0.88-1.03 | 0.80 | 0.66-0.98 | 0.82 | 0.73-0.93 |
| Age-appropriate diet |  |  |  |  |  |  |  |  |  |  |
| No (=0) | 1 |  | 1 |  | 1 |  | 1 |  | 1 |  |
| Yes (=1) | 1.03 | 0.96-1.10 | 1.02 | 0.90-1.16 | 1.06 | 0.98-1.16 | 1.20 | 0.85-1.69 | 0.93 | 0.69-1.24 |
| Maternal height, cm |  |  |  |  |  |  |  |  |  |  |
| <145.0 (=0) | 1 |  | 1 |  | 1 |  | 1 |  | 1 |  |
| 149.9-145.0 (=1) | 0.90 | 0.86-0.94 | 0.82 | 0.75-0.89 | 1.00 | 0.96-1.04 | 0.79 | 0.62-1.00 | 0.86 | 0.70-1.06 |
| 154.9-150.0 (=1) | 0.78 | 0.74-0.81 | 0.67 | 0.61-0.73 | 1.42 | 1.27-1.58 | 0.62 | 0.53-0.87 | 0.75 | 0.61-0.92 |
| ≥155.0 (=1) | 0.66 | 0.62-0.691 | 0.48 | 0.42-0.56 | 1.33 | 1.20-1.48 | 0.49 | 0.36-0.68 | 0.61 | 0.49-0.75 |
| Women involve in income |  |  |  |  |  |  |  |  |  |  |
| No (=0) | 1 |  | 1 |  | 1 |  | 1 |  | 1 |  |
| Yes (=1) | 0.98 | 0.94-1.01 | 1.01 | 0.94-1.08 | 1.02 | 0.98-1.07 | 1.002 | 0.81-1.23 | 0.94 | 0.83-1.06 |
| Sex of the children |  |  |  |  |  |  |  |  |  |  |
| Male (=0) | 1 |  | 1 |  | 1 |  | 1 |  | 1 |  |
| Female (=1) | 0.95 | 0.92-0.98 | 0.96 | 0.89-1.02 | 0.95 | 0.91-0.98 | 0.91 | 0.77-1.07 | 1.04 | 0.94-1.16 |
| WAZ |  |  |  |  |  |  |  |  |  |  |
| Non-underweight (=0) | 1 |  | 1 |  | 1 |  | 1 |  | 1 |  |
| Underweight (=1) | 3.64 | 3.52-3.77 | 3.84 | 3.58-4.16 | 3.59 | 3.44-3.74 | 4.07 | 3.39-4.88 | 3.01 | 2.69-3.37 |
| WHZ |  |  |  |  |  |  |  |  |  |  |
| Non-wasting (=0) | 1 |  | 1 |  | 1 |  | 1 |  | 1 |  |
| Wasting (=1) | 0.42 | 0.40-0.44 | 0.51 | 0.46-0.58 | 0.40 | 0.38-0.42 | 0.72 | 0.55-0.93 | 0.58 | 0.48-0.70 |
| BMI, in kg/m^2^ |  |  |  |  |  |  |  |  |  |  |
| Thin (<18.50) | 1 |  | 1 |  | 1 |  | 1 |  | 1 |  |
| Normal (18.50-24.99) | 0.99 | 0.96-1.03 | 0.96 | 0.88-1.03 | 1.00 | 0.96-1.04 | 0.95 | 0.78-1.17 | 1.11 | 0.98-1.06 |
| Overweight (25.00-29.99) | 0.91 | 0.87-0.95 | 0.90 | 0.81-0.99 | 0.91 | 0.86-0.97 | 0.90 | 0.70-1.15 | 0.89 | 0.77-1.02 |
| Obesity (≥30.00) | 0.99 | 0.90-1.08 | 0.99 | 0.80-1.23 | 0.95 | 0.91-0.98 | 1.13 | 0.71-1.79 | 0.98 | 0.79-1.20 |
| Child age, in month |  |  |  |  |  |  |  |  |  |  |
| 0-5 | 1 |  | 1 |  | 1 |  | 1 |  | 1 |  |
| 6-23 | 1.43 | 1.31-1.56 | 1.55 | 1.30-1.85 | 1.42 | 1.27-1.58 | 1.18 | 0.74-1.88 | 1.77 | 1.06-2.03 |
| 24-59 | 1.40 | 1.29-1.53 | 1.50 | 1.25-1.80 | 1.33 | 1.20-1.48 | 1.32 | 0.85-2.07 | 1.89 | 1.38-2.57 |
| Diarrhoea |  |  |  |  |  |  |  |  |  |  |
| No (=0) | 1 |  | 1 |  | 1 |  | 1 |  | 1 |  |
| Yes (=1) | 1.02 | 0.96-1.07 | 0.87 | 0.74-1.03 | 1.01 | 0.94-1.08 | 0.81 | 0.60-1.09 | 1.05 | 0.94-1.16 |
| Fever |  |  |  |  |  |  |  |  |  |  |
| No (=0) | 1 |  | 1 |  | 1 |  | 1 |  | 1 |  |
| Yes (=1) | 0.97 | 0.93-1.01 | 0.96 | 0.95-1.03 | 0.96 | 0.91-1.02 | 0.93 | 0.77-1.12 | 1.11 | 1.002-1.24 |
| Wealth index |  |  |  |  |  |  |  |  |  |  |
| Poorest | 1 |  | 1 |  | 1 |  | 1 |  | 1 |  |
| Poor | 0.93 | 0.90-0.97 | 0.99 | 0.98-1.08 | 0.93 | 0.88-0.97 | 0.76 | 0.60-0.95 | 1.06 | 0.93-1.21 |
| Middle | 0.87 | 0.83-0.92 | 0.90 | 0.81-1.00 | 0.88 | 0.83-0.93 | 0.66 | 0.52-0.84 | 0.97 | 0.80-1.18 |
| Fourth | 0.79 | 0.75-0.84 | 0.86 | 0.76-0.96 | 0.79 | 0.74-0.84 | 0.54 | 0.40-0.74 | 0.90 | 0.73-1.12 |
| Richest | 0.72 | 0.67-0.78 | 0.68 | 0.58-0.81 | 0.74 | 0.67-0.81 | 0.44 | 0.34-0.71 | 0.85 | 0.66-1.10 |
| Residence |  |  |  |  |  |  |  |  |  |  |
| Urban (=0) | 1 |  | 1 |  | 1 |  | 1 |  | 1 |  |
| Rural (1) | 1.01 | 0.97-1.06 | 1.03 | 0.98-1.05 | 0.99 | 0.94-1.05 | 1.19 | 1.01-1.41 | 0.94 | 0.82-1.07 |

**** p<.01, ** p<.05, * p<.1. - adjusted relative risk; RR<1 positive association; RR=1 no association; RR>1 negative association; CI - Confidence Interval*

**Appendix 7**

Latent variable scores were calculated based on factor loadings obtained from confirmatory factor analysis. The relationships between latent variables and their indicators were summarized by factor loadings, and model fit was assessed using Akaike Information Criterion (AIC) and Bayesian Information Criterion (BIC). All reported coefficients are unstandardized.

**Appendix 7a: Factor loading for the latent Social Independence**

| **Variable** | **Unstandardized**  **Co-efficient** | **SE** | **P-value** |
| --- | --- | --- | --- |
| Reading newspapers | 1 | 0 | ---- |
| Highest years of schooling | 3.03 | 0.08 | 0.000 |
| Women age at 1^st^ birth, | 6.19 | 0.14 | 0.000 |
| Women age at 1^st^ cohabitation | 6.62 | 0.15 | 0.000 |
| Age difference between husband and wife | 1.79 | 0.05 | 0.000 |
| Years of schooling difference between husband and wife | 0.47 | 0.06 | 0.000 |

AIC: 1090937; BIC: 1091082

**Appendix 7b:** Factor loading for the latent intrinsic agency

| **Variable** | **Unstandardized Co-efficient** | **SE** | **P-value** |
| --- | --- | --- | --- |
| Wife beating is justified if, |  |  |  |
| Wife goes out without telling husband | 1 | 0 | ---- |
| Wife neglects the children | 0.99 | 0.02 | 0.000 |
| Wife argues with husband | 1.19 | 0.03 | 0.000 |
| Wife refuses to have sex with husband | 0.91 | 0.02 | 0.000 |
| Wife burns food | 0.77 | 0.02 | 0.000 |

AIC: 129057.9; BIC:129185.9

**Appendix 7c:** Factor loading for the latent instrumental agency

| **Variable** | **Unstandardized Co-efficient** | **SE** | **P-value** |
| --- | --- | --- | --- |
| Person who usually decides, |  |  |  |
| Respondent’s healthcare | 1 | 0 | ---- |
| Large household purchase | 1.42 | 0.03 | 0.000 |
| Respondent visits to family/ relatives | 1.32 | 0.02 | 0.000 |

AIC: 147627.4; BIC:147704.2

**Appendix 8:**

We fitted a generalized linear mixed model with Poisson distribution, log link, and robust standard errors to estimate adjusted relative risks of stunting. The models included women’s empowerment domains (social independence, intrinsic agency, and instrumental agency), food intake, WaSH interaction terms between empowerment domains and food intake and WaSH, maternal height, women’s involvement in income-generating activities, child sex, WAZ, and WHZ.

Let

- $Y_{ij}$= stunting status of child $i$in cluster $j$
- $Y_{ij}=1$if stunted, $0$otherwise

Then the models will be :

Model 1. $Y_{ij}\sim\text{Poisson}(\mu_{ij})$

$$\log(\mu_{ij})=\beta_{0}+\beta_{1}SI_{ij}+\beta_{2}IA_{ij}+\beta_{3}INA_{ij}+\beta_{4}WASH_{ij}+\beta_{8}FI_{ij}+\beta_{9}MH_{ij}+\beta_{10}WI_{ij}+\beta_{11}SEX_{ij}+\beta_{12}WAZ_{ij}+\beta_{13}WHZ_{ij}+u_{j}$$

Model 2. $Y_{ij}\sim\text{Poisson}(\mu_{ij})$

$$\log(\mu_{ij})=\beta_{0}+\beta_{1}SI_{ij}+\beta_{2}IA_{ij}+\beta_{3}INA_{ij}+\beta_{4}WASH_{ij}+\beta_{5}(SI_{ij}\times WASH_{ij})+\beta_{6}(IA_{ij}\times WASH_{ij})+\beta_{7}(INA_{ij}\times WASH_{ij})+\beta_{8}FI_{ij}+\beta_{9}MH_{ij}+\beta_{10}WI_{ij}+\beta_{11}SEX_{ij}+\beta_{12}WAZ_{ij}+\beta_{13}WHZ_{ij}+u_{j}$$

Model 3. $Y_{ij}\sim\text{Poisson}(\mu_{ij})$

$$\log(\mu_{ij})=\beta_{0}+\beta_{1}SI_{ij}+\beta_{2}IA_{ij}+\beta_{3}INA_{ij}+\beta_{4}FI_{ij}+\beta_{5}(SI_{ij}\times FI_{ij})+\beta_{6}(IA_{ij}\times FI_{ij})+\beta_{7}(INA_{ij}\times FI_{ij})+\beta_{8}WASH_{ij}+\beta_{9}MH_{ij}+\beta_{10}WI_{ij}+\beta_{11}SEX_{ij}+\beta_{12}WAZ_{ij}+\beta_{13}WHZ_{ij}+u_{j}$$

Where:

- $SI$= social independence
- $IA$= intrinsic agency
- $INA$= instrumental agency
- $FI$= food intake
- $WASH$= WaSH facility
- $MH$= maternal height
- $WI$= women involved in income-generating activity
- $SEX$= sex of the child
- $WAZ$= weight-for-age z-score
- $WHZ$= weight-for-height z-score
- $u_{j}$= random effect for cluster/community/household, with

$$u_{j}\sim N(0,\sigma_{u}^{2})$$

Then the adjusted relative risk is: $RR=e^{\beta_{k}}$
Robust standard errors are used to correct variance misspecification.

**Reference:**

Obiageli, E.I. and A.J. Chinyere, Using Poisson Regression Model and its Application in a University System (A Case Study of University of Benin). International Journal of Research and Innovation in Applied Science, 2025. 10(8): p. 292-307.

**Appendix 9: Nutritional status of under-five children in selected population (weighted)**

| **Indicators** |  | | **Country**  **n=37620** | | | | | | | |
| --- | --- | --- | --- | --- | --- | --- | --- | --- | --- | --- |
|  | **Pooled** | | **Bangladesh** | | **India** | | **Nepal** | | **Pakistan** | |
|  | **N=37620** | | **N=6271** | | **N=26398** | | **N=1539** | | **N=3412** | |
|  | **n** | **%** | **n** | **%** | **n** | **%** | **n** | **%** | **n** | **%** |
| **Height-for-age z score** |  |  |  |  |  |  |  |  |  |  |
| Non-stunted (≥-2 SD) | 24413 | 64.89 | 4256 | 67.87 | 16986 | 64.35 | 1105 | 71.80 | 2066 | 60.55 |
| Stunted (<-2 SD) | 13207 | 35.11 | 2015 | 32.13 | 9412 | 35.65 | 434 | 28.20 | 1346 | 39.45 |
| **Weight-for-age z score** |  |  |  |  |  |  |  |  |  |  |
| Non-underweight (≥-2 SD) | 27379 | 72.78 | 4824 | 76.93 | 18684 | 70.78 | 1242 | 80.70 | 2629 | 77.05 |
| Underweight (<-2 SD) | 10241 | 27.22 | 1447 | 23.07 | 7714 | 29.22 | 297 | 19.30 | 783 | 22.95 |
| **Weight-for-height z score** |  |  |  |  |  |  |  |  |  |  |
| Non-wasting (≥-2 SD) | 31784 | 84.49 | 5725 | 91.29 | 21502 | 81.45 | 1447 | 92.72 | 3130 | 91.74 |
| Wasting (<-2 SD) | 5836 | 15.51 | 546 | 8.71 | 4896 | 18.55 | 112 | 7.28 | 282 | 8.26 |

**Appendix 10: Association between Stunting among under-five children and women’s empowerment domains and other covariates (weighted)**

| **Covariates** | **Pooled** | | **Countries** | | | | | | | |
| --- | --- | --- | --- | --- | --- | --- | --- | --- | --- | --- |
|  |  |  | **Bangladesh** | | **India** | | **Nepal** | | **Pakistan** | |
|  | **adj-RR** | **95%CI** | **adj-RR** | **95%CI** | **adj-RR** | **95%CI** | **adj-RR** | **95%CI** | **adj-RR** | **95%CI** |
| Social independence | 0.86*** | 0.83-0.90 | 0.82*** | 0.75-0.90 | 0.88*** | 0.85-0.92 | 0.92 | 0.77-1.10 | 0.75*** | 0.68-0.83 |
| Intrinsic agency | 0.99* | 0.99-1.002 | 0.98** | 0.97-0.99 | 1.00 | 0.99-1.002 | 0.97 | 0.93-1.01 | 0.99 | 0.98-1.01 |
| Instrumental agency | 0.991** | 0.98-0.993 | 0.99 | 0.97-1.01 | 0.99* | 0.97-1.00 | 0.99 | 0.96-1.03 | 0.99 | 0.96-1.01 |
| Age-appropriate diet |  |  |  |  |  |  |  |  |  |  |
| No (=0) | 1 |  | 1 |  | 1 |  | 1 |  | 1 |  |
| Yes (=1) | 0.89*** | 0.82-0.96 | 0.87** | 0.78-0.97 | 0.92** | 0.86-0.92 | 1.03 | 0.71-1.39 | 0.65*** | 0.50-0.84 |
| WaSH practice |  |  |  |  |  |  |  |  |  |  |
| No (=0) | 1 |  | 1 |  | 1 |  | 1 |  | 1 |  |
| Yes (=1) | 0.90*** | 0.87-0.93 | 0.92** | 0.86-0.99 | 0.91*** | 10.87-0.94 | 0.72*** | 0.60-0.87 | 0.81*** | 0.73-0.90 |
| Maternal height, cm |  |  |  |  |  |  |  |  |  |  |
| <145.0 (=0) | 1 |  | 1 |  | 1 |  | 1 |  | - | - |
| 149.9-145.0 (=1) | 0.89*** | 0.83-0.96 | 0.81*** | 0.74-0.88 | 0.92*** | 0.88-0.97 | 0.79*** | 0.63-0.99 | - | - |
| 154.9-150.0 (=1) | 0.75*** | 0.68-0.82 | 0.65*** | 0.60-0.72 | 0.78*** | 0.74-0.82 | 0.67*** | 0.52-0.85 | - | - |
| ≥155.0 (=1) | 0.62*** | 0.55-0.71 | 0.48*** | 0.42-0.55 | 0.66*** | 0.62-0.70 | 0.46*** | 0.34-0.63 | - | - |
| Women involve in income |  |  |  |  |  |  |  |  |  |  |
| No (=0) | 1 |  | 1 |  | 1 |  | 1 |  | 1 |  |
| Yes (=1) | 1.04*** | 1.02-1.07 | 1.07** | 1.003-1.15 | 1.04* | 0.99-1.08 | 1.25** | 1.04-1.52 | 1.00 | 0.88-1.12 |
| Sex of the children |  |  |  |  |  |  |  |  |  |  |
| Male (=0) | 1 |  | 1 |  | 1 |  | 1 |  | 1 |  |
| Female (=1) | 0.96** | 0.94-0.98 | 0.96 | 0.90-1.03 | 0.95** | 0.91-0.99 | 0.90 | 0.77-1.06 | 1.01 | 0.91-1.12 |
| WAZ |  |  |  |  |  |  |  |  |  |  |
| Non-underweight (=0) | 1 |  | 1 |  | 1 |  | 1 |  | 1 |  |
| Underweight (=1) | 3.72*** | 3.55-3.91 | 4.02*** | 3.73-4.32 | 3.68*** | 3.53-3.84 | 4.32*** | 3.62-5.17 | 3.36*** | 3.03-3.73 |
| WHZ |  |  |  |  |  |  |  |  |  |  |
| Non-wasting (=0) | 1 |  | 1 |  | 1 |  | 1 |  | 1 |  |
| Wasting (=1) | 0.42*** | 0.38-0.47 | 0.51*** | 0.45-0.57 | 0.40*** | 0.38-0.43 | 0.66*** | 0.51-0.84 | 0.53*** | 0.44-0.64 |

**** p<.01, ** p<.05, * p<.1. - adjusted relative risk; RR<1 positive association; RR=1 no association; RR>1 negative association; CI - Confidence Interval*

Model 1: HAZ**= S**ocial independence+ Intrinsic agency+ Instrumental agency+ Food intake+ WaSH+ Other covariates

*Pooled: Chi-square 9807; Prob>chi2 0.000; AIC 45436; BIC 45546; VIF 1.46*

*Bangladesh: Chi-square 2378; Prob>chi2 0.000; AIC 7230; BIC 7318; VIF 1.35*

*India: Chi-square 6585; Prob>chi2 0.000; AIC 32596; BIC 32702; VIF 1.44*

*Nepal: Chi-square 513; Prob>chi2 0.000; AIC 1610; BIC 1679; VIF 1.60*

*Pakistan: Bangladesh: Chi-square 827; Prob>chi2 0.000; AIC 3908; BIC 3967; VIF 1.10.* (-) Not in the model;

**Appendix 11: Interaction effects between SWPER domains and WASH practice in household on stunting of under-five children**

| **Covariates** | **Pooled** | | **Countries** | | | | | | | |
| --- | --- | --- | --- | --- | --- | --- | --- | --- | --- | --- |
|  |  |  | **Bangladesh** | | **India** | | **Nepal** | | **Pakistan** | |
|  | **adj-RR** | **95%CI** | **adj-RR** | **95%CI** | **adj-RR** | **95%CI** | **adj-RR** | **95%CI** | **adj-RR** | **95%CI** |
| Social independence | 0.95* | 0.90-1.006 | 0.95 | 0.84-1.08 | 0.89*** | 0.84-0.96 | 1.16 | 0.88-1.53 | 0.92 | 0.79-1.07 |
| Intrinsic agency | 0.98*** | 0.97-0.99 | 0.98* | 0.96-1.002 | 0.99 | 0.98-1.003 | 0.98 | 0.91-1.05 | 1.009 | 0.78-1.02 |
| Instrumental agency | 0.99 | 0.97-1.01 | 0.99 | 0.96-1.02 | 1.004** | 0.98-1.02 | 1.01 | 0.93-1.08 | 1.02 | 0.97-1.02 |
| WaSH facility |  |  |  |  |  |  |  |  |  |  |
| No (=0) | 1 |  | 1 |  | 1 |  | 1 |  | 1 |  |
| Yes (=1) | 0.89*** | 0.83-0.92 | 0.83*** | 0.74-0.92 | 0.91*** | 0.88-0.95 | 0.67*** | 0.55-0.82 | 0.71*** | 0.61-0.83 |
| Social Independence* WaSH | 0.92*** | 0.86-0.99 | 0.76*** | 0.64-0.91 | 0.97* | 0.90-1.06 | 0.72* | 0.51-1.02 | 0.75*** | 0.61-0.92 |
| Intrinsic agency*WaSH | 1.007 | 0.99-1.01 | 0.99 | 0.96-1.02 | 1.01* | 0.99-1.02 | 0.99 | 0.91-1.07 | 0.98 | 0.95-1.01 |
| Instrumental agency*WaSH | 0.98* | 0.99-1.03 | 0.99 | 0.96-1.03 | 0.97* | 0.95-1.002 | 0.97 | 0.94-1.06 | 0.96 | 0.90-1.02 |
| Age-appropriate diet |  |  |  |  |  |  |  |  |  |  |
| No (=0) | 1 |  | 1 |  | 1 |  | 1 |  | 1 |  |
| Yes (=1) | 0.87*** | 0.83-0.92 | 0.87** | 0.78-0.97 | 0.92** | 0.86-0.99 | 1.002 | 0.74-1.34 | 0.66*** | 0.51-0.85 |
| Maternal height, cm |  |  |  |  |  |  |  |  |  |  |
| <145.0 (=3) | 1 |  | 1 |  | 1 |  | 1 |  | - |  |
| 149.9-145.0=2) | 0.90*** | 0.86-0.93 | 0.81*** | 0.75-0.88 | 0.92*** | 0.88-0.97 | 0.80* | 0.63-1.008 | - | - |
| 154.9-150.0 (=1) | 0.76*** | 0.73-0.79 | 0.65*** | 0.60-0.72 | 0.78*** | 0.74-0.82 | 0.67*** | 0.52-0.85 | - | - |
| ≥155.0 (=0) | 0.64*** | 0.61-0.67 | 0.48*** | 0.42-0.55 | 0.66*** | 0.62-0.79 | 0.47*** | 0.34-0.63 | - | - |
| Women had employment |  |  |  |  |  |  |  |  |  |  |
| No (=0) | 1 |  | 1 |  | 1 |  | 1 |  | 1 |  |
| Yes (=1) | 1.004 | 0.97-1.03 | 1.07** | 1.004-1.15 | 1.04* | 0.99-1.08 | 1.26** | 1.04-1.52 | 1.01 | 0.90-1.14 |
| Sex of the children |  |  |  |  |  |  |  |  |  |  |
| Male (=0) | 1 |  | 1 |  | 1 |  | 1 |  | 1 |  |
| Female (=1) | 0.96** | 0.93-0.99 | 0.96 | 0.90-1.03 | 0.95** | 0.91-0.99 | 0.91 | 0.77-1.07 | 1.009 | 0.91-1.11 |
| WAZ |  |  |  |  |  |  |  |  |  |  |
| Non-underweight (=0) | 1 |  | 1 |  | 1 |  | 1 |  | 1 |  |
| Underweight (=1) | 3.77*** | 3.65-3.90 | 4.003*** | 3.71-4.31 | 3.68*** | 3.53-3.84 | 4.32*** | 3.61-5.16 | 3.34*** | 3.008-3.70 |
| WHZ |  |  |  |  |  |  |  |  |  |  |
| Non-wasting (=0) | 1 |  | 1 |  | 1 |  | 1 |  | 1 |  |
| Wasting (=1) | 0.42*** | 0.40-0.45 | 0.51*** | 0.45-0.57 | 0.40*** | 0.38-0.43 | 0.66*** | 0.51-0.84 | 0.53*** | 0.44-0.64 |

*** p<.01, ** p<.05, * p<.1. RR- relative risk; RR<1 positive association; RR=1 no association; RR>1 negative association; CI - Confidence Interval

Model 2: HAZ= social independence+ intrinsic agency+ instrumental agency+ food intake+ WaSH+ social independence* WaSH+ intrinsic Agency* WaSH+ Instrumental Agency*WaSH+ other covariates.

Pooled: Chi-square 9889; Prob>chi2 0.000; AIC 45434; BIC 45570; VIF 1.56

Bangladesh: Chi-square 2381; Prob>chi2 0.000; AIC 7232; BIC 7339; VIF 1.76. India: Chi-square 6658; Prob>chi2 0.000; AIC 32597; BIC 32728; VIF 1.54

Nepal: Chi-square 519; Prob>chi2 0.000; AIC 1613; BIC 1699; VIF 1.61. Pakistan: Bangladesh: Chi-square 850; Prob>chi2 0.000; AIC 3905; BIC 3983; VIF 1.45. (-) Not in the model

**Appendix 12: Risk of stunting among under five children with interaction between age SWPER domains and age-appropriate food intake in South Asia (mixed effect GLM, random effect for country, and fixed effect for predictors)**

| **Covariates** | **Pooled** | | **Countries** | | | | | | | |
| --- | --- | --- | --- | --- | --- | --- | --- | --- | --- | --- |
|  |  |  | **Bangladesh** | | **India** | | **Nepal** | | **Pakistan** | |
|  | **adj-RR** | **95%CI** | **adj-RR** | **95%CI** | **adj-RR** | **95%CI** | **adj-RR** | **95%CI** | **adj-RR** | **95%CI** |
| Social independence | 0.85*** | 0.82-0.89 | 0.80*** | 0.73-0.89 | 0.87*** | 0.83-0.91 | 0.89 | 0.73-1.08 | 0.74*** | 0.67-0.83 |
| Intrinsic agency | 0.99** | 0.99-1.00 | 0.98 | 0.93-1.00 | 1.00 | 0.99-1.007 | 0.96 | 0.93-1.007 | 1.001 | 0.98-1.01 |
| Instrumental agency | 0.98** | 0.98-0.99 | 0.99 | 0.97-1.01 | 0.98** | 0.97-0.99 | 0.99 | 0.96-1.03 | 0.99 | 0.96-1.02 |
| WaSH facility |  |  |  |  |  |  |  |  |  |  |
| No (=0) | 1 |  | 1 |  | 1 |  | 1 |  | 1 | 1 |
| Yes (=1) | 0.90*** | 0.87-0.93 | 0.92** | 0.86-0.99 | 0.91*** | 0.87-0.94 | 0.72*** | 0.59-0.87 | 0.81*** | 0.73-0.90 |
| Age-appropriate diet |  |  |  |  |  |  |  |  |  |  |
| No (=0) | 1 |  | 1 |  | 1 |  | 1 |  | 1 |  |
| Yes (=1) | 0.89*** | 0.84-0.95 | 0.94 | 0.81-1.08 | 0.91** | 0.85-0.98 | 0.95 | 0.68-1.33 | 0.60*** | 0.44-0.83 |
| Social Independence* Age-appropriate diet | 1.12** | 1.07-1.17 | 1.16 | 0.89-1.50 | 1.13* | 0.99-1.30 | 1.25 | 0.82-1.90 | 1.21 | 0.81-1.82 |
| Intrinsic agency* Age-appropriate diet | 0.98 | 0.97-1.00 | 0.99 | 0.94-1.03 | 0.99 | 0.97-1.01 | 1.08 | 0.93-1.24 | 0.92** | 0.86-0.99 |
| Instrumental agency*Age-appropriate diet | 1.03** | 1.01-1.05 | 1.02 | 0.96-1.09 | 1.03 | 0.99-1.07 | 0.96 | 0.81-1.14 | 1.04 | 0.92-1.19 |
| Maternal height, cm |  |  |  |  |  |  |  |  |  |  |
| <145.0 (=3) | 1 |  | 1 |  | 1 |  | 1 |  |  |  |
| 149.9-145.0=2) | 0.89*** | 0.83-0.96 | 0.81*** | 0.74-0.88 | 0.92*** | 0.88-0.97 | 0.78** | 0.62-0.98 | - | - |
| 154.9-150.0 (=1) | 0.75*** | 0.68-0.82 | 0.65*** | 0.59-0.72 | 0.78*** | 0.74-0.82 | 0.66*** | 0.52-0.84 | - |  |
| ≥155.0 (=0) | 0.62*** | 0.55-0.71 | 0.48** | 0.41-0.55 | 0.66*** | 0.62-0.70 | 0.46*** | 0.34-0.63 | - | - |
| Women had employment |  |  |  |  |  |  |  |  |  |  |
| No (=0) | 1 |  | 1 |  | 1 |  | 1 |  | 1 |  |
| Yes (=1) | 1.04** | 1.02-1.07 | 1.07** | 1.002-1.15 | 1.04* | 0.99-1.08 | 1.25** | 1.03-1.51 | 0.99 | 0.88-1.11 |
| Sex of the children |  |  |  |  |  |  |  |  |  |  |
| Male (=0) | 1 |  | 1 |  | 1 |  | 1 |  | 1 |  |
| Female (=1) | 0.96** | 0.94-0.98 | 0.96 | 0.90-1.03 | 0.95** | 0.91-0.99 | 0.90 | 0.76-1.06 | 1.01 | 0.91-1.11 |
| WAZ |  |  |  |  |  |  |  |  |  |  |
| Non-underweight (=0) | 1 |  | 1 |  | 1 |  | 1 |  | 1 |  |
| Underweight (=1) | 3.72*** | 3.54-3.90 | 4.01*** | 3.73-4.32 | 3.68*** | 3.53-3.83 | 4.32*** | 3.61-5.16 | 3.36*** | 3.03-3.73 |
| WHZ |  |  |  |  |  |  |  |  |  |  |
| Non-wasting (=0) | 1 |  | 1 |  | 1 |  | 1 |  | 1 |  |
| Wasting (=1) | 0.42*** | 0.38-0.47 | 0.51*** | 0.45-0.57 | 0.40*** | 0.38-0.43 | 0.66*** | 0.51-0.84 | 0.53*** | 0.44-0.63 |

**** p<.01, ** p<.05, * p<.1. - adjusted relative risk; RR<1 positive association; RR=1 no association; RR>1 negative association; CI - Confidence Interval*

*Model 3: HAZ= social independence+ intrinsic agency+ instrumental agency+ food intake+ social independence* food intake+ intrinsic Agency* food intake+ Instrumental Agency*food intake+ WaSH+ other covariates*

*Pooled: Chi-square 9873; Prob>chi2 0.000; AIC 45431; BIC 45567; VIF 4.37*

*Bangladesh: Chi-square 2383; Prob>chi2 0.000; AIC 7235; BIC 7343; VIF 3.25; India: Chi-square 6624; Prob>chi2 0.000; AIC 32596; BIC 32727; VIF 4.63*

*Nepal: Chi-square 519; Prob>chi2 0.000; AIC 1615; BIC 1700; VIF 5.21; Pakistan: Bangladesh: Chi-square 828; Prob>chi2 0.000; AIC 3908; BIC 3967; VIF 5.86.* (-) Not in the model
